# Supplementary material for: DNA contamination within recombinant adeno-associated virus preparations correlates with decreased CD34+ cell clonogenic potential
Source: Mol Ther Methods Clin Dev. 2024 Sep 12;32(4):101334. doi: 10.1016/j.omtm.2024.101334 (PMC11460252; doi:10.1016/j.omtm.2024.101334)
Supplement: Document S1. Figures S1–S4 and Tables S1–S4 [file mmc1.pdf]

OMTM, Volume *32*

Supplemental information

DNA contamination within recombinant adeno-associated virus preparations correlates with decreased CD34^{+} cell clonogenic potential

Christopher R. Luthers, Sung-Min Ha, Annika Mittelhauser, Marco Morselli, Joseph D. Long, Caroline Y. Kuo, Zulema Romero, and Donald B. Kohn

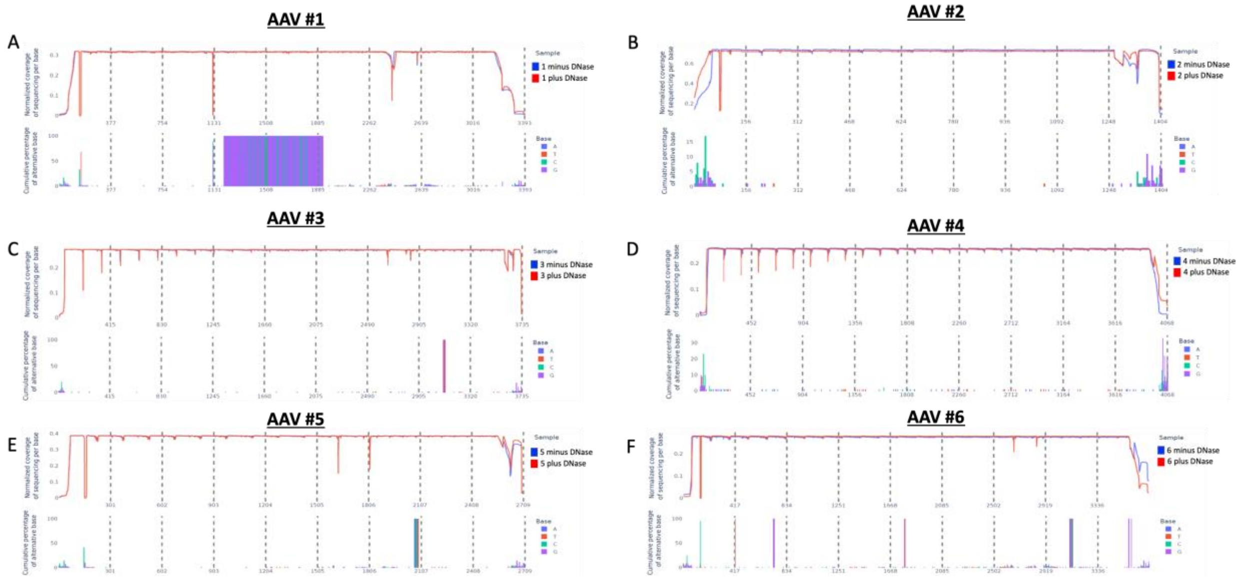

Figure S1: Read Coverage and Single Nucleotide Variants Across the AAV Genome. (Top) Coverage of sequencing reads across the rAAV genome was normalized by dividing the number of reads aligned to each base and diving that by the total coverage of all bases which mapped to the AAV genome. (Bottom) Total percentage of alternative nucleotide variants (A=blue, T=red, C=green, G=purple) uncovered from each base call compared to reference rAAV genomic sequences. (A)=AAV #1, (B)=AAV #2, (C)=AAV #3, (D)=AAV #4, (E)=AAV #5, (F)=AAV #6

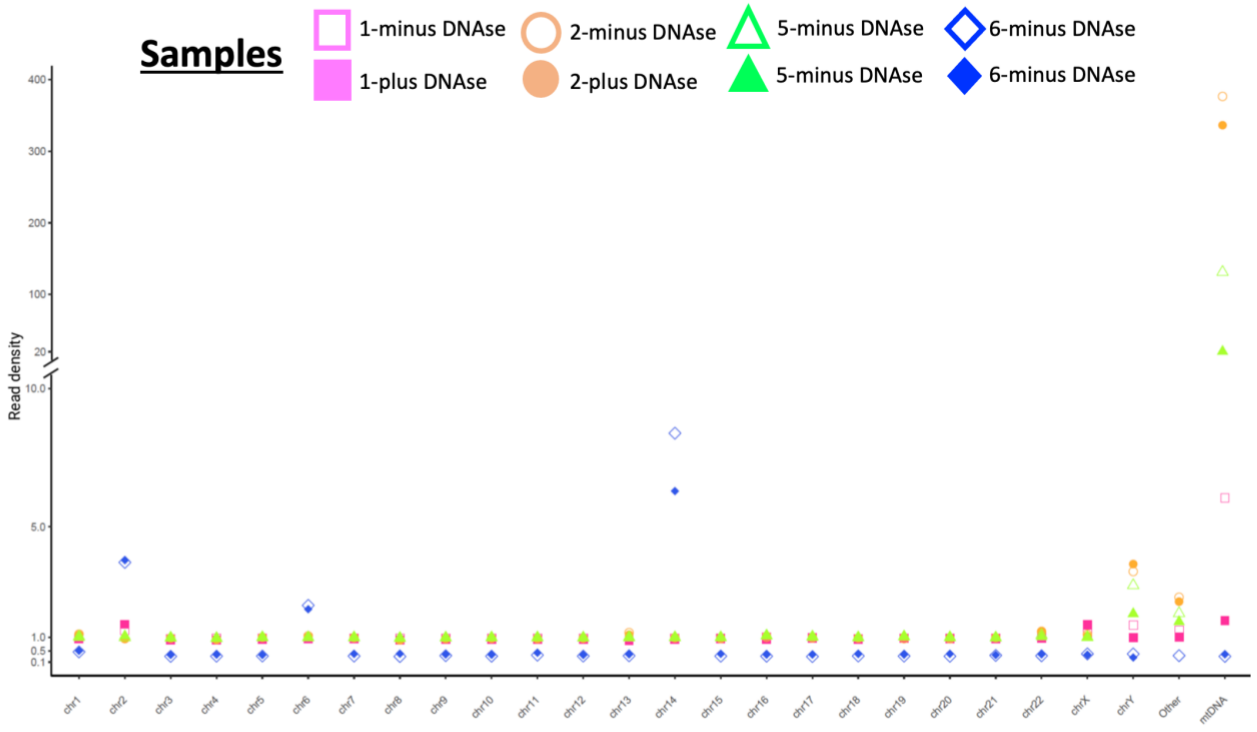

Figure S2: Distribution of Genomic DNA Contaminants Across Human Genome Chromosomes. For the four preps which were packaged using human cells, read depth of contaminants from genomic DNA was mapped to each human chromosome. Read density of 1=random distribution, read density 2 = 2 fold enrichment**.**

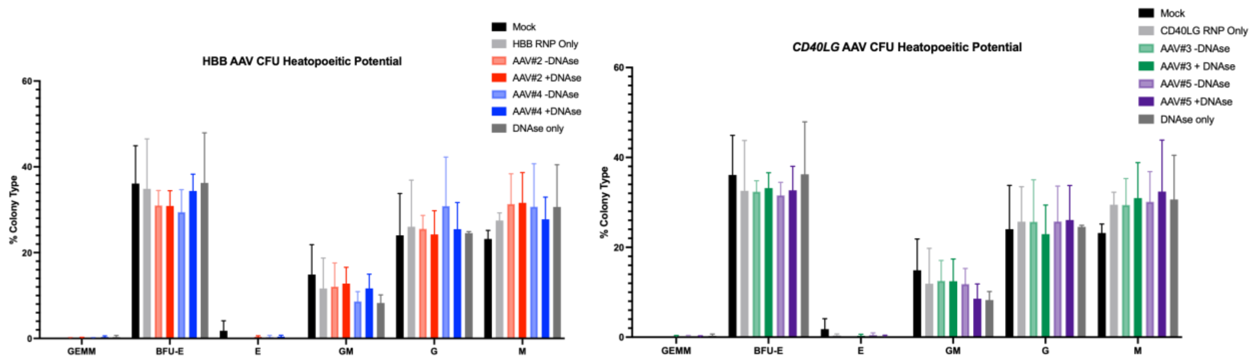

Figure S3: Progenitor analysis of CFU assay. At 24 h post-EP, PBSCs were harvested, counted by trypan blue exclusion, and plated in methylcellulose to perform the colony-forming unit (CFU) assay. At 12–14 days after plating the cells in methylcellulose, colonies were enumerated. For each plate, the percentage of cells growing was obtained as the total number of colonies counted normalized to the number of cells plated per dish for rAAV6 preps targeting the HBB (A) and CD40LG (B) loci, respectively. n = 3 independent experiments from three different PBSC donors. Error bars, mean ± SD.

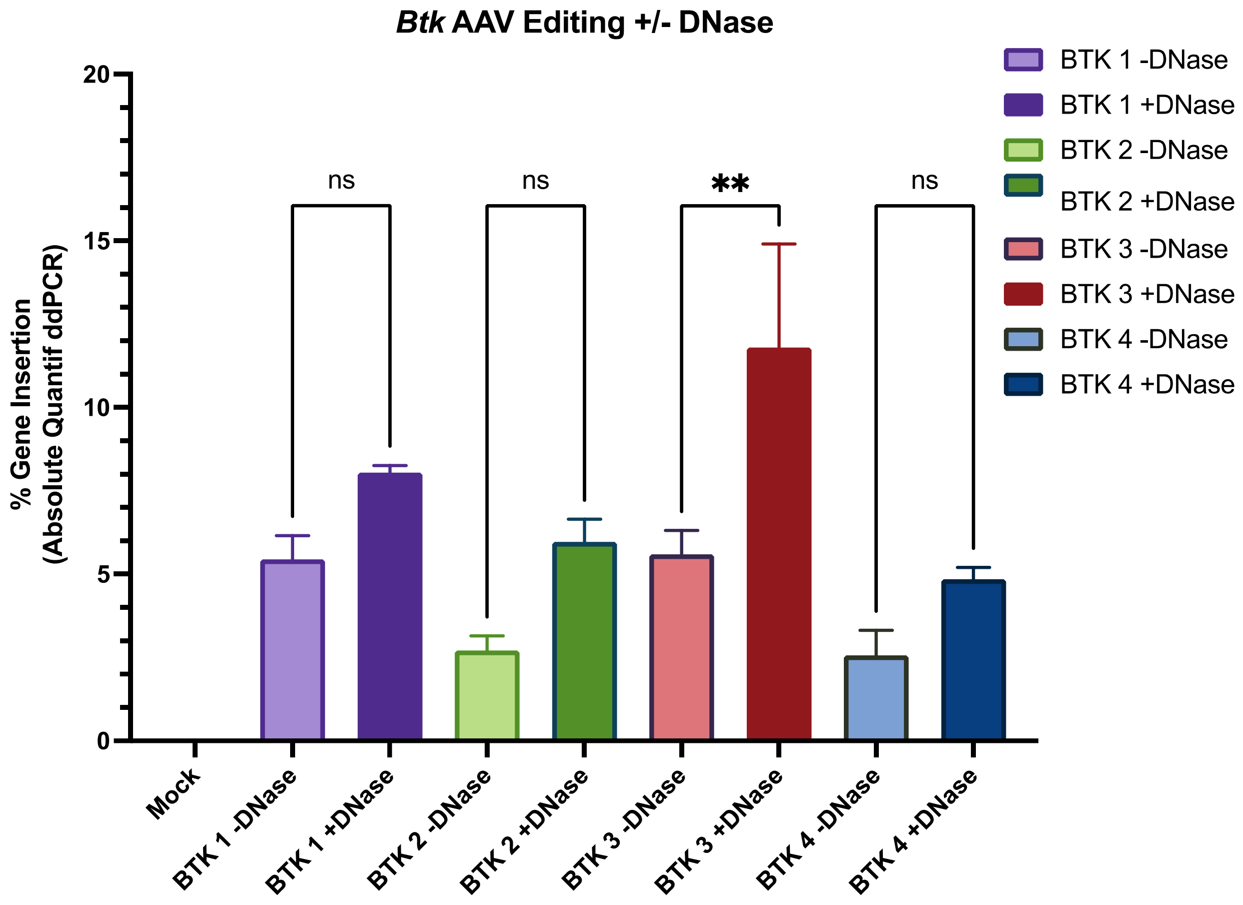

Figure S4: DNAse pretreatment increases editing in alternative cell types and genomic loci. Bone marrow derived lineage depleted Lin- cells HSPC were harvested from Btk/Tec-/- mice cells. Following 48-hour prestimulation, lin- cells were then electroporated with Cas9-RNP targeting the endogenous *Btk* locus and transduced with 4 rAAV6 vectors +/- DNAse treatment (Table 1). 5 days post transduction, gDNA of Lin- cells was harvested followed by droplet digital PCR analysis of *Btk* site-specific editing analysis. Values indicate the percentage of DNA sequences with successful insertion of corrective *Btk* cDNA sequences. N=3 independent murine Lin- transductions.

Table S1: Read Quality and Depth of NGS Analysis. PHRED quality scores and number of reads for each NGS run analyzed. “Treatment” refers to absence or presence of DNAse treatment of the rAAV6 preps.

Sample Name	Treatment	Mean read quality	Number of reads
1-minus	-	27.61	8,499,170
		27.72	8,499,170
1-plus	+	28.44	9,938,328
		27.67	9,938,328
2-minus	-	28.66	30,718,308
		27.50	30,718,308
2-plus	+	28.50	24,433,069
		27.56	24,433,069
3-minus	-	28.19	21,862,350
		26.99	21,862,350
3-plus	+	27.47	25,443,374
		26.74	25,443,374
4-minus	-	28.04	5,787,053
		26.85	5,787,053
4-plus	+	28.57	23,258,440
		27.12	23,258,440
5-minus	-	27.66	10,846,160
		26.89	10,846,160
5-plus	+	27.85	6,195,556
		27.09	6,195,556
6-minus	-	26.49	3,920,919
		27.77	3,920,919
6-plus	+	26.33	2,142,433
		26.68	2,142,433

Table S2: Comparing the Read Distribution of ITR for NGS Analysis in Merged AAV-Vector Backbone Reference Sequences. To further analyze the read quality and distribution of NGS reads across the ITR, Contavect analysis was conducted using two methods: one with a merged reference sequence containing the entire vector plasmid (AAV + ITR sequences) or using unique reference sequences for the rAAV genome and AAV plasmid backbone sequence. Values shown above are raw number of reads in the ITR's, the AAV sequence, and the percentage of total reads which come from the ITR.

	Separated AAV and Backbone references			Merged AAV and Backbone references		
	reads in ITR	reads in AAV	% reads in ITR	reads in ITR	reads in AAV	% reads in ITR
1-minus	340,316	5,495,240	4.28%	344,342	5,495,222	4.33%
	351,381	5,347,083	4.42%	356,109	5,347,032	4.47%
1-plus	523,613	8,414,450	5.5%	531,204	8,415,850	5.58%
	544,833	8,331,851	5.73%	554,020	8,334,639	5.82%
2-minus	7,403	327,111	0.02%	13,314	343,967	0.04%
	8,034	321,643	0.03%	13,525	337,355	0.05%
2-plus	5,529	282,079	0.02%	12,344	282,189	0.05%
	5,506	279,138	0.02%	12,309	279,200	0.05%
3-minus	611,739	16,912,460	2.96%	619,981	16,909,716	3%
	330,099	16,429,882	1.6%	346,920	16,429,493	1.68%
3-plus	883,705	22,695,634	3.72%	892,696	22,682,335	3.76%
	395,254	20,938,698	1.67%	412,161	20,938,388	1.74%
4-minus	101,523	5,311,944	1.84%	38,930	5,291,672	0.71%
	75,475	5,248,768	1.37%	40,588	5,245,309	0.74%
4-plus	287,258	21,713,704	1.27%	144,689	21,652,884	0.64%
	302,498	21,185,477	1.34%	155,085	21,165,785	0.69%
5-minus	130,254	4,687,617	1.28%	134,373	4,689,318	1.32%
	197,388	4,451,614	1.94%	204,585	4,452,895	2.01%
5-plus	144,484	4,920,014	2.5%	148,053	4,921,760	2.57%
	218,092	4,752,140	3.78%	224,893	4,753,502	3.9%
6-minus	43,815	2,659,202	1.22%	43,960	2,659,247	1.22%
	38,888	2,315,550	1.08%	39,087	2,315,603	1.09%
6-plus	26,946	1,663,936	1.41%	27,021	1,663,961	1.41%
	25,801	1,556,942	1.35%	25,926	1,556,994	1.35%

Table S3: Absolute Read Numbers of DNA Products Within AAV Preps. The amount of reads for each DNA contaminant as they map to known contaminant sequences. Numbers represent total individual reads.

Reference name	1-minus	1-plus	2-minus	2-plus	5-minus	5-plus	6-minus	6-plus
Phi X174	2	4	12	4	1	1	3	2
rAAV genome	10,858,317	16,767,045	649,015	561,952	9,146,508	9,680,613	4,979,995	3,223,010
Plasmid backbone	440,435	748,995	128,276	100,832	132,442	95,575	33,515	19,737
Helper plasmid	479,620	814,580	782,540	741,113	252,222	134,868	235,163	139,504
Human genome	312,984	74,242	51,800,765	40,761,803	5,368,041	996,459	33,695	18,260
Vigene Rep Cap	25,633	48,246	197,455	166,776	48,399	18,266	11,890	6,760
Unmappe d	3,798,663	576,216	6,389,869	5,422,208	5,368,749	611,770	1,895,955	422,821
Total	15,915,654	1,902,9328	59,947,932	47,754,688	20,316,362	11,537,552	7,190,216	3,830,094

Reference name	3-minus	3-plus	4-minus	4-plus
Phi X174	18	17	1	6
rAAV genome	33,356,896	43,648,328	10,560,924	42,901,282
Plasmid backbone	0	0	0	0
Full plasmid	317,144	324,340	14,195	74,387
Baculovirus	83,027	73,175	32,192	188,441
Virovek Rep Cap	1,904	1,069	570	2,979
Sf-9	27,108	24,599	24,576	89,726
Unmapped	7,520,875	3,377,918	406,552	1,971,951
Total	41,306,972	47,449,446	11,039,010	45,228,772

Table S4. Example Table for DNase Treatment of rAAV6 Vectors. Sample calculation sheet for pretreatment of rAAV

			DNAse Calculation						
**Condition**	**uL AAV**	**uL ATP**	**uL Baseline DNAse**	**uL Plasmid safe DNAse**	**uL H20**	**Total Volume**	**uL 10x buffer**	**Total uL before STOP**	**uL 10x STOP**
**2 +DNase**	10.83	20	10	4	0	44.83	4.48	49.32	4.93
**2 -DNase**	10.83	20	0	0	14.00	44.83	4.48	49.31	4.93
**4 +DNase**	6.16	20	10	4	4.67	44.83	4.48	49.31	4.93
**4 -DNase**	6.16	20	0	0	18.67	44.83	4.48	49.31	4.93
**3 +DNase**	3.23	20	10	4	7.60	44.83	4.48	49.31	4.93
**3 -DNase**	3.23	20	0	0	21.60	44.83	4.48	49.31	4.93
**5 +DNAse**	4.92	20	10	4	5.91	44.83	4.48	49.31	4.93
**5 -DNAse**	4.92	20	0	0	19.91	44.83	4.48	49.31	4.93
**DNAse only**	0.00	20	10	4	10.83	44.83	4.48	49.31	4.93
