# Supplementary material for: DNA contamination within recombinant adeno-associated virus preparations correlates with decreased CD34+ cell clonogenic potential
Source: Mol Ther Methods Clin Dev. 2024 Sep 12;32(4):101334. doi: 10.1016/j.omtm.2024.101334 (PMC11460252; doi:10.1016/j.omtm.2024.101334)
Supplement: Document S2. Article plus supplemental information [file mmc2.pdf]

Molecular Therapy
Methods & Clinical Development
Original Article

DNA contamination within recombinant adeno-associated virus preparations correlates with decreased CD34$^+$ cell clonogenic potential

Christopher R. Luthers,[1,2] Sung-Min Ha,[3] Annika Mittelhauser,[2] Marco Morselli,[4] Joseph D. Long,[2] Caroline Y. Kuo,[2] Zulema Romero,[2] and Donald B. Kohn[2]

[1]Molecular Biology Interdepartmental Program, University of California, Los Angeles (UCLA), Los Angeles, CA, USA; [2]Department of Microbiology, Immunology, and Molecular Genetics, UCLA, Los Angeles, Los Angeles, CA, USA; [3]Department of Integrative Biology and Physiology, UCLA, Los Angeles, CA, USA; [4]Department of Molecular, Cell, and Developmental Biology, UCLA, Los Angeles, CA, USA

Recombinant adeno-associated viruses (rAAV) are promising for applications in many genome editing techniques through their effectiveness as carriers of DNA homologous donors into primary hematopoietic stem and progenitor cells (HSPCs), but they have many outstanding concerns. Specifically, their biomanufacturing and the variety of factors that influence the quality and consistency of rAAV preps are in question. During the process of rAAV packaging, a cell line is transfected with several DNA plasmids that collectively encode all the necessary information to allow for viral packaging. Ideally, this process results in the packaging of complete viral particles only containing rAAV genomes; however, this is not the case. Through this study, we were able to leverage single-stranded virus (SSV) sequencing, a next-generation sequencing-based method to quantify all DNA species present within rAAV preps. From this, it was determined that much of the DNA within some rAAV preps is not vector-genome derived, and there is wide variability in the contamination by DNA across various preps. Furthermore, we demonstrate that transducing CD34$^+$ HSPCs with preps with higher contaminating DNA resulted in decreased clonogenic potential, altered transcriptomic profiles, and decreased genomic editing. Collectively, this study characterized the effects of DNA contamination within rAAV preps on CD34$^+$ HSPC cellular potential.

INTRODUCTION

Due to promising results in clinical trials, recombinant adeno-associated virus (rAAV) vectors have been used to treat genetic diseases by gene therapy.[1,2] One common application of rAAV is to provide homologous donor sequences for gene editing in hematopoietic stem and progenitor cells (HSPCs). Despite the efficacy of rAAV vectors as DNA donors, outstanding questions remain regarding the quality of biomanufacturing of the rAAV preps.[3] Specifically, variations in intrinsic characteristics such as full versus empty capsid ratios, viral titer, and purity of rAAV genomes within preps are not fully characterized and are the focus of this study.[4] Furthermore, the role of these factors in the progenitor potential of transduced HSPCs remains unanswered.

To generate recombinant AAV vector preps, a packaging cell line is transfected with several DNA plasmids containing the information for generation of the viral capsid, accessory proteins, and rAAV genome containing the DNA of interest between the inverted-terminal repeats.[5] Ideally, this process allows for the production of only complete vectors, with pure AAV genomes making up the entire DNA compartment; however, this is not the case. Contaminating DNA may come from the genome of the producer cells, helper and packaging plasmids, or foreign exogenous DNA contamination, and it has the potential to elicit direct toxicity to HSPCs.[4] Transduction of HSPCs with preps containing high levels of DNA contamination has the potential to activate Toll-like receptors and induce interferons (IFNs) and alternative intracellular DNA-response signaling pathways.[6] This activation can result in cellular-wide transcriptomic variation and resultant decreases in transduced stem cell potential.[7] These resultant alterations can decrease not only rAAV homology-directed repair (HDR)-mediated genomic editing via alterations in cell-cycle status but also HSPC clonogenic potential, a critical proxy for *in vivo* stem cell engraftment.[8]

In this study, we characterized significant differences in contaminating DNA among rAAV preparations from different sources and correlated our findings with differences in transduced HSPC clonogenic potential, acute cellular transcriptional responses, and genome editing outcomes. We treated six previously studied rAAV preps from three different manufacturers, with and without DNase treatment, to allow for the quantification of contaminating DNA inside versus outside of the viral capsid. Following DNase treatment (or not), total DNA was isolated and analyzed by Illumina next-generation sequencing (NGS). In parallel, CD34$^+$ HSPCs were edited and transduced with these rAAV preparations, followed by measurement of

Received 1 May 2024; accepted 5 September 2024;
https://doi.org/10.1016/j.omtm.2024.101334.

Correspondence: Donald B. Kohn, MD, Department of Microbiology, Immunology, and Molecular Genetics, UCLA, 3163 Terasaki Life Science Bldg., 610 Charles E. Young Drive, Los Angeles, CA 90095, USA.
E-mail: dkohn1@mednet.ucla.edu

© 2024 The Author(s). Published by Elsevier Inc. on behalf of The American Society of Gene and Cell Therapy. This is an open access article under the CC BY license (http://creativecommons.org/licenses/by/4.0/).

Table 1. Vector prep demographics

Prep no.	Locus	Manufacturer no.	Producer cell	Packaging system	Purification	Titer, genome copies/mL	Benzonase pretreatment?
1	*CD40LG*	1	HEK293T	triple transfection	ultra-centrifugation	1.20e+13	no
2	*HBB*	1	HEK293T	triple transfection	IDX	3.52e+13	no
3	*CD40LG*	2	Sf9	baculovirus	CsCl	2.00e+13	yes
4	*HBB*	2	Sf9	baculovirus	CsCl	2.11e+13	yes
5	*CD40LG*	1	HEK-293T	triple transfection	IDX	1.32 $CD34^{+}$e+13	no
6	*HBB*	3	HEK293T	triple transfection	IDX	2.07e+12	no
BTK 1	*mBtk*	2	Sf9	baculovirus	CsCl	2.00e+13	yes
BTK 2	*mBtk*	1	HEK293T	triple transfection	IDX	1.33e+13	no
BTK 3	*mBtk*	2	Sf9	baculovirus	CsCl	2.00e+13	yes
BTK 4	*mBtk*	1	HEK293T	triple transfection	IDX	8.19e+13	no

Table of genomic locus, manufacturer, producer cell, packaging system, purification method, and titer of 10 rAAV6 vector preps characterized. Manufacturer 1, Vigene Biosciences; manufacturer 2, Virovek Biosciences; manufacturer 3, University of North Carolina Vector Core.

viability and the methylcellulose-based colony-forming unit (CFU) assay to measure clonogenic potential. We also performed Reverse Transcriptase quantitative PCR (RT-qPCR) analysis for changes in expression of a panel of genes associated with cell cycle, acute immune response, and apoptosis.

Collectively, this study identifies the presence of contaminating DNA within rAAV preps as a critical quality characteristic affecting HSPC cellular potential that should be strongly considered when selecting a manufacturing source for AAV vectors for HSPC editing.

RESULTS

Analysis of DNA contaminants in AAV preps

To characterize the role of contaminant DNA within AAV preps, six rAAV preps were chosen from three distinct manufacturers produced by different packaging and purification methods (Table 1). Preexisting AAV preps were used for the analysis as it had been seen that these preps targeting the exact same genomic locus resulted in differences in viability, editing, and clonogenicity within given $CD34^{+}$ HSPCs. It was critical for the experimental design to have rAAV preps targeting different loci and using different AAV packaging strategies. Four of the six preps (1, 2, 5, and 6) used standard triple transfection of HEK293T cells for vector production, but AAV number 1 was produced in a different manufacturing facility. The two other preps, AAV numbers 3 and 4, used baculovirus-mediated infection of Sf9 insect cells to produce AAVs (Table 1). All listed titers are based on the given titers from the product sheet shipped from the commercial manufacturers. The range of manufacturers, production systems, and genomic targets allowed for increased confidence in results and the ability to compare multiple variables in one comprehensive study.

AAV vector preps (2×10^{11}) were treated with a combination of both baseline-ZERO endonuclease and plasmid safe exonuclease (Biosearch Technologies), to ensure complete digestion of DNA present outside of the viral capsids. Following this, total DNA was isolated, following a viral lysis using Qiagen cell lysis solution and the DNA precipitation method, from all six rAAV preps treated with and without DNase. All isolated DNA was then denatured, and single-stranded DNA (ssDNA) was captured using the SRSLY Nanoplus ssDNA→cDNA kit, followed by phosphorylation of template DNA, adapter ligation, and unique molecular identifier (UMI) indexing adapter addition. The final library containing DNA from all six preps (+/−DNase treatment) was then run using paired-end NovaSeq next-generation DNA sequencing. Because the sequences of all plasmids required for transfection of the cells to produce the AAV as well as the entire genome of the human 293T and insect Sf9 cells are known, each sequencing read was then mapped to all known contaminants, and the percentages of each mapped read were quantified[9] (Tables 2 and 3).

In analyzing the percentages of each read being mapped to known sequences, any DNA present within the rAAV preps that is not from the rAAV genome is considered "contaminant" DNA. It was striking to see the high variation of the "purity" levels within the AAV genomes as %rAAV genomes among all the DNA species in the analyzed preps (Tables 2 and 3). For example, AAV number 1 only had 1% of rAAV genome within its prep, whereas AAV number 4 had as high as 95% pure AAV genomic DNA. Additionally, there were clear differences in the purity of rAAV genomes depending on the manufacturer, with manufacturer 2, who used Sf9 cells and a baculovirus system clearly having the highest purity of preps as compared to manufacturers 1 and 3. This is likely due to the purification process and methods that were conducted to purify the preps. For example, AAV number 1 was a "crude" prep; the packaging cell line was collected and subjected to differential centrifugation for purification with no CsCl or iodixanol gradient ultracentrifugation (IDX) addition. Preps from manufacturer 2 underwent CsCl purification followed by density gradient-mediated isolation of rAAV preps. Also noteworthy, all rAAV preps had low levels of residual *Escherichia coli* genomic DNA, likely indicating *E. coli* genomic DNA carryover from nucleic acid plasmid preps required for the transfection of packaging cell lines. Importantly, our read quality and depth were of

Table 2. Percentages of DNA populations in rAAV preps obtained by NGS—HEK293T cell packaged AAVs

	AAV no.							
	1	1	2	2	5	5	6	6
DNase	–	+	–	+	–	+	–	+
rAAV genome, %	1.08	1.18	45.02	83.91	69.26	84.15	68.22	88.11
Human genome, %	86.41	85.36	26.42	8.64	0.47	0.48	1.97	0.39
Helper plasmid, %	1.55	1.25	1.24	1.17	3.27	3.64	3.01	4.28
Plasmid backbone, %	0.21	0.21	0.65	0.83	0.47	0.52	2.77	3.94
Rep-Cap plasmid, %	0.33	0.35	0.24	0.16	0.17	0.18	0.16	0.25
E. coli genome, %	1.06	0.97	2.13	0.87	1.79	1.47	2.33	0.67
Unmapped DNA, %	9.56	10.38	24.40	4.43	24.58	9.57	21.54	2.36

Data represent percentages of individual DNA species that contribute to the total DNA analysis for rAAV6 preps, which were packaged using HEK293T cells. Unmapped DNA is DNA sequences that did not map to any of the known DNA sequences listed above.

sufficient levels (Tables S1–S3). Additionally, read coverage and accuracy was relatively well maintained throughout the entire rAAV genome sequence (Figure S1).

The relative increase in rAAV genomes upon DNase reduction of contaminating human cellular DNA indicated that the majority of contaminating DNA was outside of the AAV viral capsid, not contained within the virion, allowing for a potential DNase-mediated "cleanup" of AAV preps prior to transduction. In contrast to contamination with human genomic DNA, the levels of the contaminating helper plasmid, Rep-Cap, and plasmid backbone DNA in the preps did not change much with DNase treatment, suggesting that these species were primarily intra-virion. Additionally, human genomic DNA contaminants did not disproportionately map to any specific chromosome (Figure S2). Due to the high percentage of unmapped reads in specific preps, lowQ score and unmapped DNA sequences were analyzed via NCBI BLAST. Interestingly, the DNA mapped to a wide variety of contaminants; however, a large percentage of unmapped reads partially aligned to the *E. coli* genome, indicating potentially even higher levels of contaminant DNA carryover from bacterial cloning and bacterial plasmid DNA preparations (Supplemental file available upon request).

Table 3. Percentages of DNA populations in rAAV preps obtained by NGS—Sf9 cell packaged AAVs

	AAV no.			
	3	3	4	4
DNase treatment	–	+	–	+
rAAV genome, %	80.75	91.99	95.67	94.85
Sf9 cell genome, %	0.07	0.052	0.22	0.2
Baculovirus, %	0.2	0.15	0.29	0.42
Plasmid backbone, %	0.77	0.68	0.13	0.16
Rep-Cap plasmid, %	0.005	0.002	0.0052	0.0066
E. coli genome, %	0.73	0.61	0.83	0.47
Unmapped DNA, %	17.48	6.51	2.85	3.89

Data represent percentages of individual DNA species that contribute to the total DNA analysis for rAAV6 preps, which were packaged using Sf9 cells. Unmapped DNA is DNA sequences that did not map to any of the known DNA sequences listed above.

Collectively, this analysis showed that production method, manufacturer, purification method, and DNase treatment play critical roles in the purity of a rAAV prep. Given this promising analysis, we sought to further characterize the role of the purity of rAAV preps on clonogenic potential, transcriptional response, and genome editing within transduced CD34$^+$ HSPCs.

Analysis of effects of AAV preps on human CD34$^+$ HSPC activity

For autologous hematopoietic stem cell gene therapy, CD34$^+$ HSPCs are harvested from a patient, followed by *ex vivo* editing of the cells. The patient then may receive myeloablative conditioning before transplantation to facilitate engraftment of edited HSPCs. Due to their critical role in populating the entire hematopoietic system, autologous HSPCs must maintain their clonogenic and multi-lineage differentiation potential as this allows for the regeneration of a fully functional hematopoietic/immune system.[10] Despite their widespread use, it has been observed that rAAVs can directly negatively impact CD34$^+$ HSPC clonogenic potential upon transduction.[11–13] While these studies examined various mechanisms causing the adverse effects of AAV on HSPC, the role of contaminating DNA in AAV preps in reducing HSPC clonogenic and engraftment potential has not been directly characterized.[4]

Knowing the purity of each of our preps, we transduced CD34$^+$ HSPCs from healthy donors (HDs) with AAV preps numbered 2, 3, 4, and 5. These preps were chosen as they include AAV targets for site-specific gene correction at two distinct genomic loci: β-globin (*HBB*) and CD40-ligand (*CD40LG*). These genes are relevant to sickle cell disease and X-linked hyper-IgM (immunoglobulin M) syndrome, respectively, both of which are known gene therapy clinical targets.[12,14,15] Additionally, these preps have very distinct profiles in their rAAV genome purity, with AAV preps numbers 2 and 5 being

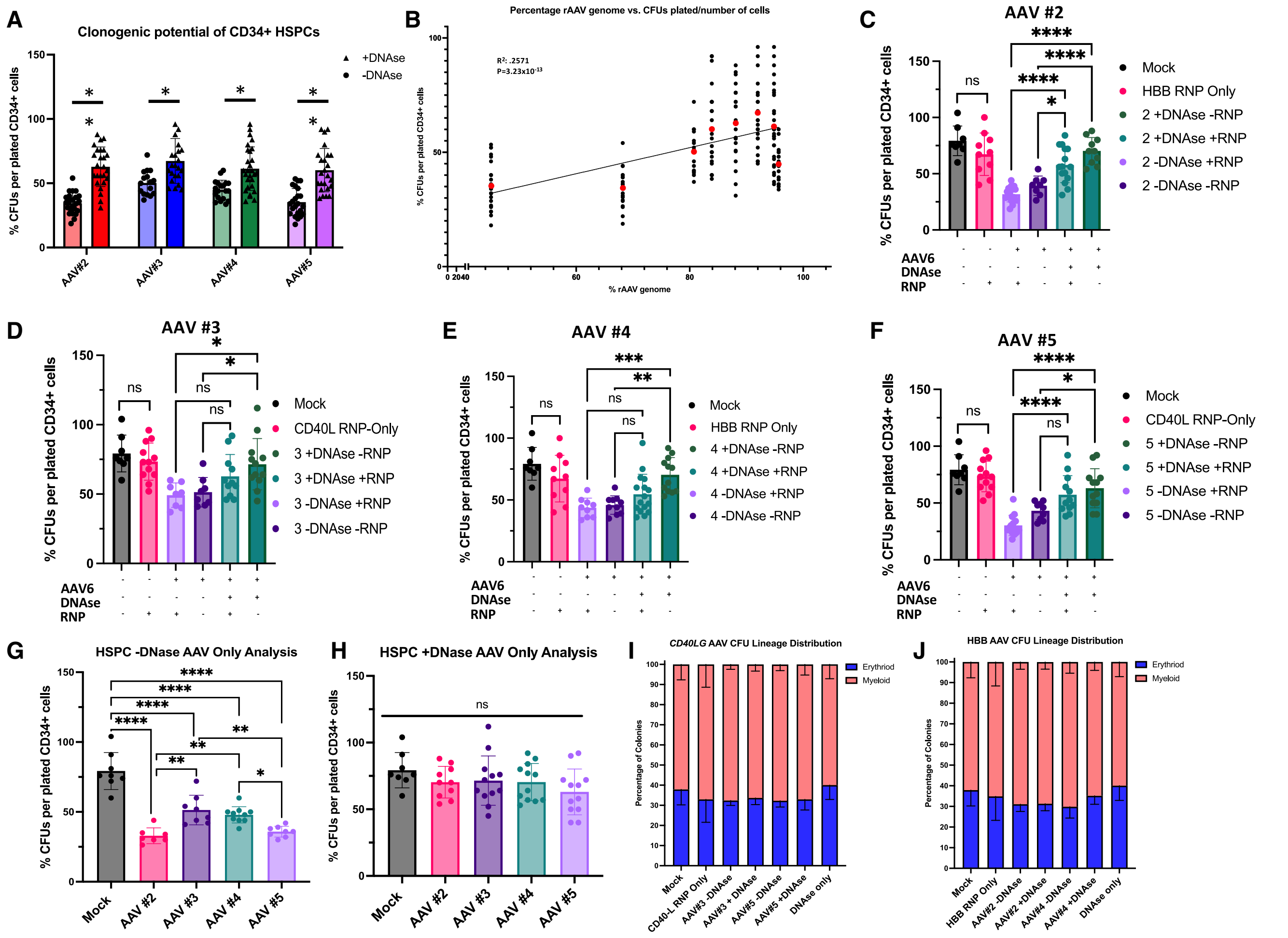

Figure 1. DNAse pretreatment of AAV6 increases CD34 clonogenic potential

HD CD34$^+$ peripheral blood HSPCs were either untransduced (mock) or transduced with rAAV at an MOI of 1e−5 (preps numbers 3 and 5) or 5e−5 (preps numbers 2 and 4) with or without RNP complex containing gRNA and Cas9 protein. CD34$^+$ cells were plated at different concentrations of methylcellulose (STEMCELL Technologies, catalog no. 04435), allowed to grow for 14 days, and individual colonies quantified and characterized. The data shown above are percentages of colonies grown in each plate per the total amount of CD34$^+$ cells plated. (A) Analysis of individual rAAV6 preps +/− DNase displays increases in colonies grown for all preps +DNase. (B) Data of %CFUs/plated cells was plotted against the percentage of the rAAV genome for each given prep displaying a trendline of increased colonies grown as purity of rAAV6 genome increase. (C–F) Data of percentage of colonies grown was plotted for each rAAV6 prep +/− DNase and +/− RNP complex. The charts below highlight the presence or absence of AAV6, DNase, and RNP. (G and H) Data of percentage of colonies grown was plotted for all four preps versus mock control +/– DNase treatment with AAV-only treatment. Preps from this analysis did not receive RNP. (I and J) Percentage of erythroid versus myeloid progenitors analyzed was plotted for CD40L (I) and HBB (J) preps. $N = 3$ individual HSPC donors. Error bars represent standard deviation. *$p < 0.05$; **$p < 0.01$; ***$p < 0.0005$; ****$p < 0.0001$.

"dirtier" preps, in that DNase treatment significantly increased the purity of the preps, and AAV preps numbers 3 and 4 being "cleaner" preps, and DNase has a less profound impact on the purity of these rAAV preps. HD CD34$^+$ HSPCs were electroporated with and without a ribonucleoprotein (RNP) complex containing a guide RNA (gRNA) and recombinant Cas9 protein targeting the *HBB* or *CD40LG* locus. After electroporation, the HSPCs were transduced with rAAV preps containing a corrective copy of the gene endogenous to the target locus, with or without prior DNase treatment of the rAAV. This allowed determination of the role of the RNP transfection, AAV transduction, and DNase treatment of rAAV preps on HSPC clonogenic potential, both individually and in combination. Following transduction of HD HSPCs with various combinations of RNP, AAV, and DNase, edited cells were placed in methylcellulose media and grown for 2 weeks, followed by a morphological analysis of colonies.

For each AAV prep, DNase treatment improved progenitor clonogenic potential as measured by a higher percentage of CFUs grown per the number of CD34$^+$ cells plated (Figure 1). Interestingly, the AAV numbers 3 and 4, in which DNase treatment displayed a much smaller increase in purity of AAV genome, showed a less

significant decrease in clonogenic potential as measured by CFUs, compared to the "dirtier" preps AAV numbers 2 and 5 ($p < 0.05$ vs. $p < 0.01$), correlating contaminating DNA and decreased clonogenic potential (Figure 1A).

To determine whether there was a global correlation between clonogenic potential and purity of AAV genomes for all preps analyzed, the purity of AAV genome calculated from the NGS analysis as %rAAV DNA among all DNA in a prep was plotted against the percentages of colonies grown for each of the preps, with and without DNase treatment. A positive trendline highlights that as purity of rAAV genomes increased within a given prep, so did the clonogenic potential of transduced CD34$^+$ cells ($R^2 = 0.2571$, $p = 3.23 \times 10^{-13}$) (Figure 1B).

Each AAV preparation was transfected/transduced into CD34$^+$ cells with a combination of RNP, rAAV6, and DNase to determine the role of each of the editing reagents on clonogenic potential. CD34$^+$ cells were again analyzed via CFU assay for all of the conditions (Figures 1C–1F). Of the reagents, the RNP alone had minimal effects on colony formation, whereas the rAAV6 preps without DNase treatment alone clearly significantly decreased colony formation (Figure 1G), as has been characterized extensively.[11–13] Also interestingly, DNase pretreatment of rAAV6 preps prior to transduction consistently improved the clonogenic potential of edited CD34$^+$ cells.

By analyzing the HSPCs that were transduced with AAV, and given no RNP, the trend became increasingly apparent: with no DNase treatment, clonogenic potential was significantly lower than that of the mock condition, with discernible differences between the preps relative to the levels of contaminant DNA. CD34$^+$ cells transduced by preps numbers 2 and 5, which had higher levels of contaminant DNA, have lower clonogenic potential relative to those transduced by preps numbers 3 and 4, which have lower levels of contaminant DNA (Figure 1G). When all preps were DNase treated, however, there were significant increases in the numbers of colonies formed, with more uniformity of the clonogenic potential across all four preps, aligning with the decreases in contaminant DNA from all preps with DNase treatment (Figure 1H). This further highlights the role of contaminant DNA within rAAV preps and the decreasing clonogenic potential of HSPCs.

Not only was it critical to quantify the effects of contaminant DNA on total clonogenic potential of the HSPCs but also the role of contaminants in potential hematopoietic lineage production was characterized. From morphological analysis of HSPCs, the percentages of myeloid and erythroid lineages from the CFU assay were quantified, as were the percentages of total erythroid versus myeloid progenitors (Figures 1I, 1J, and S3). RNP, rAAV6, and DNase treatment of HSPCs had no significant effect on lineage potential and did not result in any lineage skewing of edited cells, regardless of condition. Collectively, there is a clear correlation between increased DNA contamination within rAAV6 preps and decreased overall clonogenic potential, with no effect on lineage production of progenitor cells.

Analysis of effects of AAV preps on CD34$^+$ HSPC viability

After discovering novel and significant differences in the clonogenic potential of HSPCs transduced with AAVs of various contaminant levels, we sought to characterize how these differences affect cellular health and genome editing outcomes. In analyzing the viability of transduced HSPCs at days 1, 3, and 5 post-transduction, two trends were evident. First, all AAV-transduced cell populations showed a significant drop in viability 1 day post-transduction, compared to control cells not treated with AAV. The second evident trend was that DNase pretreatment resulted in a nonsignificant trend of increased cell viability of transduced HSPCs with all preps. Cells had a relatively higher viability of ∼70% with rAAV preps with higher purity—AAV numbers 3 and 4 (Figures 2B and 2C)—compared to cells exposed to the preps with lower purity and higher contaminant DNA, AAV numbers 2 and 5 (Figures 2A and 2D), with viability around 60%. Importantly, it was also confirmed that the DNase treatment alone, with DNase added to the cells without AAV, did not impact viability and conflate our results.

Analysis of effects of AAV preps on CD34$^+$ HSPC gene editing outcomes

We performed site-specific insertion of cDNA carried by AAV vectors into CD34$^+$ HSPCs using Cas9-mediated double-stranded DNA (dsDNA) break and HDR. When analyzing the site-specific genome editing of one of the genomic loci, *CD40LG* transduction with AAV number 3, which has higher rAAV genome purity, displayed much higher levels of DNA editing ($p < 0.001$) as compared to AAV number 5 (non-significant or ns) (Figure 3A). Additionally, DNase pretreatment increased site-specific HDR efficiency with both AAV numbers 3 and 5.

Investigating editing at the second genomic locus, *HBB*, DNase pretreatment of the AAV donors also increased HDR efficiency of both preps numbers 2 and 4 (Figure 3B). The rate of increase was more significant for prep number 2 ($p < 0.02$) when treated with DNase than for number 4 (ns), which correlates with the more significant increase in rAAV purity from DNase treatment of prep number 2 (Table 1). The beneficial effect of treating AAV donors with DNase also held true in a different cell line (murine lineage-negative cells) and genomic locus (*Btk*) with other sets of AAV vectors (Table 1), providing additional evidence of DNase-mediated increases in site-specific genome integration (Figure S4).

In summary, these data have shown a definitive role for contaminant DNA within rAAV preps on the acute viability and site-specific HDR-mediated genomic editing of HSPCs, with higher levels of contaminant DNA resulting in increased acute cytotoxicity and decreased levels of HDR, mostly alleviated by pre-treatment with DNase.

Analysis of transcriptional effects in CD34$^+$ HSPC from AAV preps

Despite these promising results and the clear inverse correlation between DNA contamination of AAV preps and colony-forming

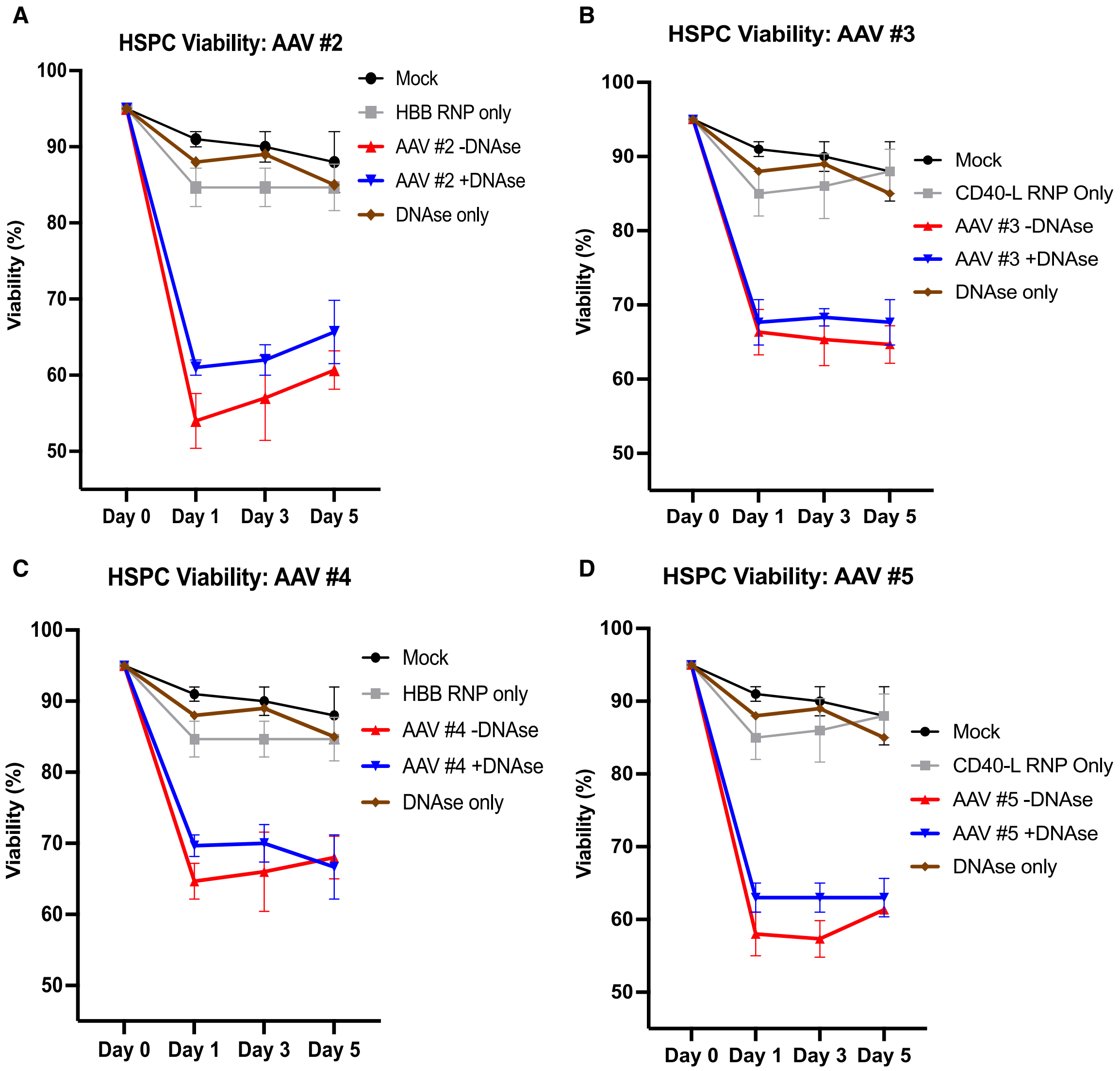

Figure 2. DNase treatment increases HSPC viability post-rAAV transduction

(A–D) CD34+ HSPCs were transduced with four rAAV6 vectors +/− DNAse treatment. Following transduction, viability was measured via trypan blue exclusion 1, 3, and 5 days post-transduction. AAV conditions received Cas9 RNP + AAV6. “RNP only” conditions received only Cas9 RNP. “DNAse only” condition received only equivalent DNase concentration as “AAV + DNase condition.” Mock condition received no DNAse, RNP, or rAAV6. *N* = 3 individual HSPC donors. Error bars represent standard deviation.

potential of transduced CD34+ HSPCs, the underlying mechanism behind this correlation was still to be characterized. To study this further, the transcriptional effects were analyzed. RNA was extracted from the same cell populations that were edited for Figure 3 at 24 h post-electroporation, followed by reverse transcription and RT-qPCR analysis of a panel of genes that has been well characterized for its role in cellular alterations following electroporation: dsDNA break formation, AAV transduction, and intracellular foreign DNA recognition.[16–19] Genes were also chosen that are associated with known cellular processes that could directly affect HSPC clonogenic potential: apoptosis, cell cycle, inflammation, and IFN signaling.[20–23] All RNA expression levels were normalized using the ΔΔCT method to a β-actin housekeeping gene, followed by normalization to a mock sample that did not receive any editing reagents.

Fold change increases in cellular expression levels of indicated transcripts are displayed in a heatmap, with the highest increases in expression levels shown in red (Figure 4A). Overall analysis of the panel of genes showed large-scale alterations in transcriptional patterns that were dependent on the editing reagents that were delivered to HSPCs. Within the larger heatmap, specific bolded sections

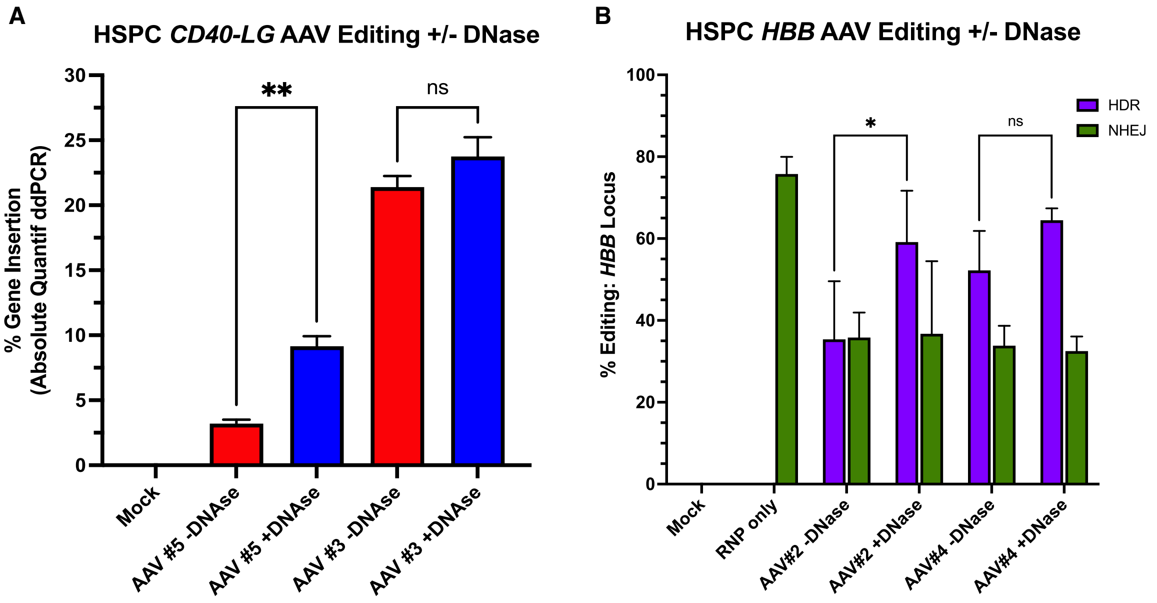

Figure 3. DNase treatment increases rAAV-mediated site-specific editing post-transduction

(A and B) CD34+ HSPCs were transduced with four rAAV6 vectors +/− DNAse treatment. Following transduction: (A) 5 days post-editing, genomic DNA (gDNA) of HSPCs was harvested, followed by ddPCR analysis of *CD40LG* site-specific editing analysis. Values indicate percentage of DNA sequences with successful insertion of corrective *CD40LG* cDNA sequences. (B) Five days post-editing, gDNA of HSPCs was harvested, followed by MiSeq-PE NGS analysis of HDR and non-homologous end joining (NHEJ). HDR rates represent rAAV6-mediated insertion of SCD mutation into the *HBB* locus of wild-type cells. $N = 3$ individual HSPC donors. Error bars represent standard deviation. *$p < 0.05$; **$p < 0.01$.

represent strikingly descriptive representations of the transcriptional effects of DNase treatment on the CD34+ HSPC state. Interestingly, pro-apoptotic gene *FAS* is downregulated in each condition upon DNase treatment, providing a direct link between DNase treatment and *FAS* gene regulation. As previously characterized, DNase treatment slightly increases the viability of edited HSPCs (Figure 2), which could be explained by a downregulation of *FAS* expression, a claim that requires further study.

The bolded first two columns to the left represent effects from AAV prep number 2, which had the largest increase in rAAV DNA when treated with DNase (Table 1). In comparing the transcriptional signatures induced by this rAAV +/− DNase, there were global decreases in almost every gene signature associated with cellular stress, cell-cycle arrest, and apoptosis from the DNase-treated prep, indicating a direct role of contaminant DNA on increased CD34+ cellular stress from AAV.

The bolded third horizontal row, indicating the gene expression of *FAS*, a key regulator of programmed cell death,[24] shows decreased expression from cells treated with each of the four preps that had been treated with DNase. This indicates that the removal of contaminant DNA within these preps resulted in a reduction in *FAS* expression. The bolded fourth horizontal row shows the relative expression of *CDKN1A*, encoding p21^{Cip1}, a regulator of cell-cycle arrest,[25] known to be upregulated upon AAV transduction.[12,13] Interestingly, DNase treatment of the rAAV preps reduced *CDKN1A* expression in most preps, indicating that contaminating DNA within rAAV preps may contribute, at least partially, to the known p21 upregulation upon rAAV transduction of HSPCs.

To further examine the key role of contaminating DNA within rAAV preps on transcriptional signatures within CD34+ HSPCs, expression data were extrapolated and displayed in individual graphs for two genes: *BAX* and *TNF-α*. BAX is a proapoptotic executioner protein capable of inducing apoptosis via mitochondrial interaction and cytochrome *c* release.[26] TNF-α (tumor necrosis factor α) is a key proinflammatory cytokine that is involved in immune cell recruitment and inflammation.[27]

When analyzing both *BAX* and TNF-α gene expression in RNA from HSPCs transduced with rAAV +/− DNase treatment, their mRNA levels were upregulated by AAV numbers 2 and 5 (the two AAV preps with high levels of contaminant DNA) when transduced without DNase pretreatment (Figures 4B, 4E, 4F, and 4I). However, when these AAV preps were treated with DNase, there was a significant reduction in proapoptotic gene expression in transduced CD34+ cells. Interestingly, when analyzing the two preps with much lower contaminating DNA, AAV numbers 3 and 4, there was little to no significant difference in the *BAX* or *TNF-α* gene expression, with or without DNase pretreatment (Figures 4C, 4D, 4G, and 4H). This further correlated the direct role of contaminant DNA and alterations of HSPC inflammatory and apoptotic gene signatures. TNF-α gene expression was slightly increased with AAV number 3 without DNase treatment relative to with DNase treatment (Figure 4G), indicating

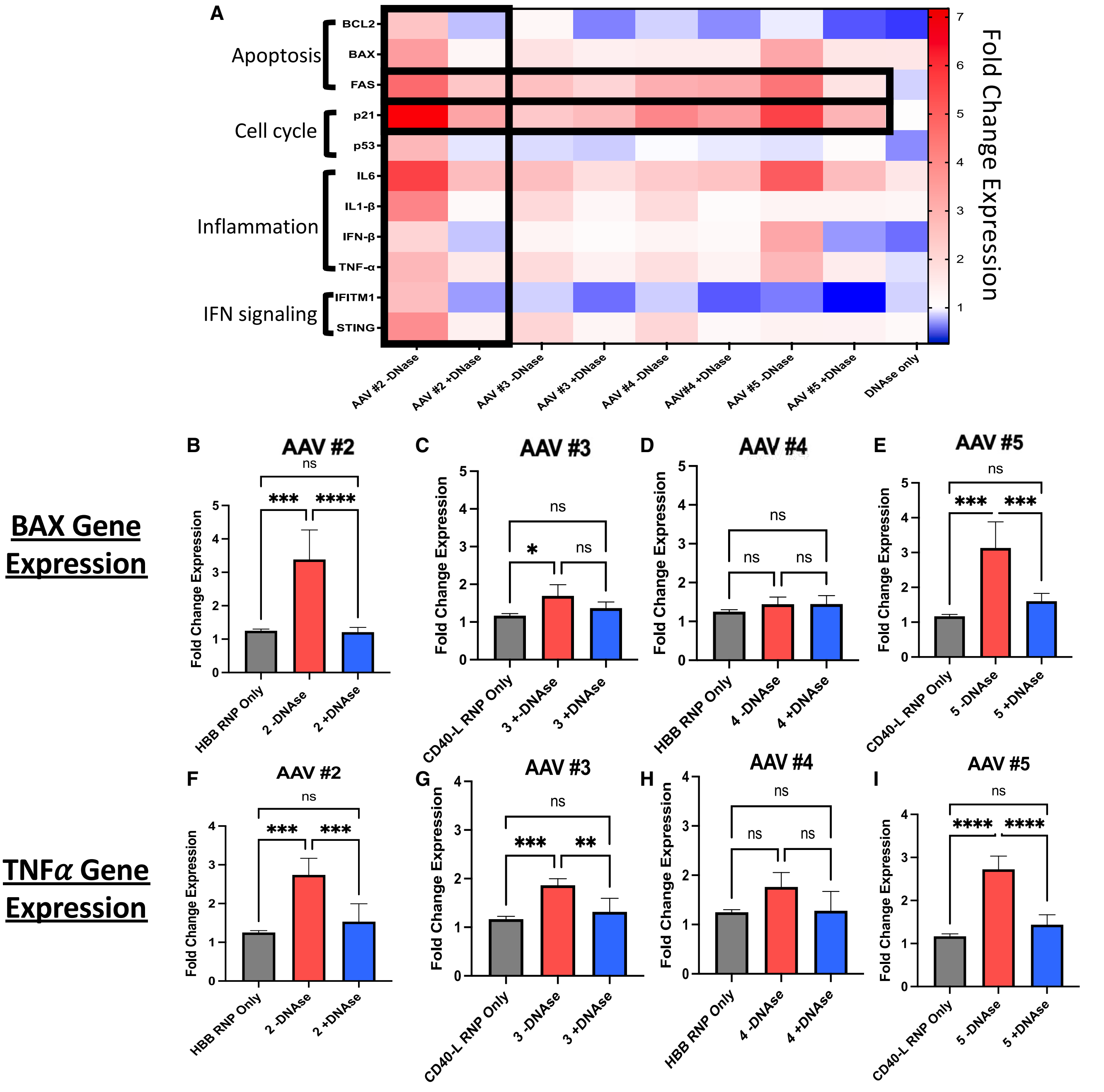

Figure 4. Contamination within rAAV preps CD34$^+$ cells results in increased apoptosis, inflammatory signaling transcripts

HD CD34$^+$ peripheral blood HSPCs were transfected with only RNP complex containing gRNA and Cas9 protein as control, or transduced with rAAV at an MOI of 1e−5 (preps numbers 3 and 5) or 5e−5 (preps numbers 2 and 4) with or without endonuclease treatment. At 24 h later, total RNA was isolated, cDNA was converted, and RNA expression analysis was completed via RT-qPCR normalized via the ΔΔCT method to β-actin gene and mock control. The data shown above are fold change in expression of the gene in title relative to mock control. (A) Heatmap of all 11 genes analyzed across all AAV +/− DNAse conditions. Colors indicate differences in fold change expression: red = upregulated, blue = downregulated. (B–E) BAX gene expression. (F–I) TNF-α gene expression. N = 3 individual donors. Error bars represent standard deviation. *$p < 0.05$; **$p < 0.001$; ***$p < 0.0005$; ****$p < 0.0001$.

either limitations of detection or intrinsic rAAV6-mediated TNF-α transcriptional effects, independent of contaminating DNA. Collectively, the RNA expression patterns from HSPCs transduced with rAAV +/− DNase pretreatment highlights a critical role of contaminant DNA on the transcriptional signatures of these cells. Collectively, we have been able to correlate the presence of contaminant

DNA within rAAV preps with the decreased viability and clonogenicity of transduced CD34+ HSPCs.

DISCUSSION

AAV preps are made through a complex process combining plasmid construction, transfection methods, and purification of viral particles.[28] Any alterations in this process will lead to inconsistencies across a variety of characteristics, including the titer of the virus, the number of particles that contain rAAV genomes, and, of course, the purity of the DNA from a given viral prep. The impetus for this study arose from our realization that different preps either from unique manufacturers or even the same manufacturer resulted in differences in HSPC viability, editing, and clonogenic potential post-transduction, but the reason was unknown. There were a variety of considerations that could impact these factors, one being the manufacturing system. As seen in the different preps that were used in this study, the packaging cell line and system can vary greatly. While different protocols will result in the successful production of virus, each of these processes will result in different DNA particles interacting with packaging cells and different potential contaminants present in each prep.[29] Another factor that must be considered when deciding on a manufacturer is the purification method. Purification typically involves ultracentrifugation and purification using a density gradient of either IDX or CsCl, both of which result in differences in the collection efficiency and purity of a given rAAV product.[30] We observed major differences among AAV6 preps between manufacturers regarding transcriptional signatures, clonogenic potential, and the viability and genome editing efficiency of CD34+ HSPCs. While each manufacturer uses a distinct packaging and purification system, a future direction would be to characterize whether it is the packaging system, purification method, or a host of other factors that results in the major differences observed in contaminant DNA across manufacturers (Table 1).

Despite the focus of this study being DNase pretreatment to improve the purity of rAAV preps, there are other strategies that could be used either in addition to DNase treatment or on their own. To minimize impurities, optimizing cell culture conditions and choosing properly characterized packaging cells can greatly impact rAAV quality.[4] Additionally, the optimization of purification strategies, including with chromatography and filtration, can efficaciously remove experiment-related impurities.[4]

While we have shown that the presence of contaminant DNA is a major factor affecting HSPC clonogenic potential and gene expression profile, it is important to acknowledge that it is not the only AAV-intrinsic factor that likely impacts HSPCs. This can be seen in the decrease in viability of all HSPCs when transduced with any rAAV prep at 1 day post-transduction (Figures 2A–2D). A variety of other factors could contribute to HSPC phenotypes, including the presence (or lack of) full versus empty AAV capsids.[31] Upon receiving a commercial rAAV prep, the main quantification of that prep is the titer, which is calculated by quantifying the amounts of rAAV genomes. This titering method, however, does not account for potential AAV capsids that do not contain rAAV genomes, and it has been shown that the presence of the AAV capsid alone can be immunogenic *in vivo*, independent of the cargo it contains.[32–34] Another major AAV-intrinsic factor is the presence of full rAAV genomes. During AAV replication, the entire rAAV genome is often not replicated, and incomplete vector genomes can be packaged within viral capsids.[35] Similar to empty capsids, incomplete vector genomes can result in improper and inconsistent transduction methods for a given prep and alterations in phenotypic readouts within a transduced cell population. Additionally, there is the possibility that contaminating RNA within AAV6 preps could also affect the characterized readouts of this study. Parallel studies pretreating AAV preps with RNAse instead of DNase could provide valuable insights into another AAV intrinsic factor affecting HSPC cellular potential. Collectively considered, there are a variety of AAV-intrinsic factors that contribute to variations in each prep. Future studies are required to determine which of these factors have significant outcomes on HSPC health and clonogenic potential and how these variations can be modified to increase consistency across various preps.

Despite being thorough, we find various limitations to our findings that can be addressed with further studies. First, one interesting question is whether DNase treatment of vectors affects infectious titers of AAV vectors. Although unlikely, future studies could be conducted to address this question by simply treating AAV6 preps with DNase and evaluating titers before and after treatment using any of the various titering methods. Second, while the CFU assay serves as an effective proxy for engraftment potential and long-term maintenance of stem cell grafts, there are systems currently available to measure the engraftment ability of edited HSPCs. There are several immunodeficient humanized mouse models capable of engrafting human CD34+ HSPCs, including the NSG, NRG, and NBSGW mouse models.[36] HSPCs edited with AAV +/− DNase could be transplanted into these mouse models, followed by the measurement of engraftment levels as a direct readout of the effect of contaminant DNA on engraftment capacity of these cells, although progenitor clonogenicity is an effective surrogate measure of engraftment.

Lastly, our RT-qPCR panel measured only the expression levels of 11 hallmark genes associated with cellular status via four biological pathways. A complete analysis of the RNA transcriptome via RNA sequencing would effectively both provide a more in-depth analysis of the additional genes associated with the pathways analyzed in this study and highlight novel alternative cellular pathways, giving a more global insight into the status of edited cells. Lastly, the analysis conducted used only 10 rAAV6 preps from three manufacturers, leaving questions around whether other AAV serotypes and other manufacturers of rAAV6 would lead to the same DNA contamination-mediated effects observed with our relatively small sample size. Future studies testing alternative AAV serotypes and unique manufacturers would provide more in-depth understanding of the role of intricacies within the rAAV production methods and their effect on HSPC status and potential.

We have been able to highlight a novel role of contaminant DNA within rAAV6 vectors and HSPC potential, a direct link that had yet to be uncovered. The findings from this study will allow for a new quality control measure of rAAV6 selection for preclinical and clinical studies, with DNase pretreatment being a consideration for the improvement of general CD34$^+$ HSPC transduction protocols. More in-depth follow-up studies will be required for the confirmation of the findings, as mentioned previously; however, this paper serves as the first connection of this specific rAAV6 feature and HSPC cellular status. Ideally, studies like this, combined with analyses of alternative AAV-intrinsic characteristics, could form a framework for a comprehensive quality control of rAAV6 preps as manufacturers and preps are selected for *in vitro* screens, *in vivo* experiments, and all studies moving gene therapy candidate vectors toward the clinic.

MATERIALS AND METHODS

CFU assay

A CFU assay was performed using Methocult H4435 enriched methylcellulose media (STEMCELL Technologies, Vancouver, BC, Canada). A total of 5,400 mobilized peripheral blood HSPCs were resuspended in Iscove's modified Dulbecco's medium + 2% fetal bovine serum, followed by serial dilutions of the cell population to achieve cell counts of 900, 300, and 75 cells, each in a total volume of 600 μL. Because the desired final cell counts were 300, 100, and 25 cells, 300 μL of each serial dilution was added to 3 mL methylcellulose aliquot, vortexed, and 1.1 mL of the total volume was plated onto a 35-mm dish with a grid in duplicate for each experimental sample using a blunt-ended needle. Dishes were then incubated at 37°C and 5% CO_2 for 14 days. Colony count and analysis were conducted using light microscopy for identification and counts.

CD34$^+$ HSPC prestimulation and electroporation

CD34$^+$ HSPC prestimulation was performed as described by Romero et al.[12,14] Electroporation of cells with editing reagents was performed as described by Romero et al. Briefly, CD34$^+$ HSPCs were electroporated with RNP complexes using BTX ECM830 Square Wave Electroporator (Harvard Apparatus) once at 250 V for 5 ms. After a 10-min rest period, cells were resuspended in 400 μL X-Vivo 15 containing cytokines and rAAV6 vector at an MOI of 1e−5 (preps numbers 3 and 5) or 5e−5 (preps numbers 2 and 4) based on methods previously optimized and described.[12,14] Cells were then rested overnight in a 37°C 5% CO_2 incubator before RNA harvest and viability analysis via trypan blue exclusion.

RT-qPCR

At 18 h post-transduction, RNA was isolated from HSPCs using the Qiagen RNeasy Plus Mini Kit (catalog no. 74136) employing manufacturer protocols, followed by quantification via nanodrop. A total of 100 ng of RNA in 10 μL was added to a reverse transcription mastermix containing 1× first stand buffer (Thermo Fisher), 10 mM DTT, 500 μM deoxynucleotide triphosphates (dNTPs), 150 ng/μL random primers (Invitrogen), 2 U/μL RNAse-OUT (Invitrogen), and 10 U/μL M-MLV reverse transcriptase (Thermo Fisher) in a total volume of 10 μL. The 20-μL RT reaction was incubated in a thermal cycler at 37°C for 60 min, 94°C for 10 min, and held at 4°C.

Following reverse transcription, an RT-qPCR reaction was conducted using PowerUp SYBR Green Master Mix (Applied Biosystems) according to the manufacturer's protocol using the ViiA7 RT-PCR system. Analysis was conducted using the standard ΔΔ method, with samples being normalized to the β-actin housekeeping gene and a mock control that received no electroporation or transduction.

ddPCR

Droplet digital PCR (ddPCR) analysis of site-specific *CD40LG* gene integration was conducted using the exact primers, probes, protocol, and assay as in Kuo et al.[14] Briefly, 5 days post-transduction of HSPCs, gDNA was harvested using the PureLink genomic DNA extraction kit (Invitrogen). Approximately 50 ng gDNA was combined in a 20-μL reaction containing in-out PCR primers to amplify both the endogenous *CD40LG* genomic locus and integrated *CD40LG* cDNA product, primers to amplify UC462 reference gene, FAM/HEX probes binding to each amplicon, EcoRV restriction enzyme, and ddPCR no dUTP ddPCR supermix (Bio-Rad).[14] Following a 1-h incubation, droplets were generated according to Bio-Rad protocols, PCR amplification was conducted in thermal cyclers, along with droplet analysis using Bio-Rad QuantaSoft ddPCR software.

DNA isolation (viruses)

DNA isolation for NGS analysis was conducted according to Lecomte et al.[9] Following DNase incubation and inactivation, DNA isolation was performed beginning by adding 300 μL of cell lysis solution (Qiagen [Qiagen cell lysis solution, RNAse A, and protein precipitation solution can be purchased together in the Qiagen Gentra Puregene Tissue Kit, catalog no. 158667]) to 220 μL rAAV +/− DNase reaction and vortexing for 10 s. Next, 20 μL proteinase K was added to all samples, inverted, and incubated at 55°C for 3 h, followed by the addition of 1.5 μL RNAse A (Qiagen), inversion, and incubation at 37°C for 15 min. Tubes were then cooled on ice for 1 min, added to 100 μL protein precipitation solution (Qiagen), and vortexed vigorously for 20 s. After 5 min of incubation on ice, samples were centrifuged at 16,000 × *g* for 5 min at 4°C. Supernatants were then transferred to new tubes, followed by an additional 300 μL isopropanol and 2 μL of 20 mg/mL glycogen (Thermo Fisher). Tubes were then mixed by inversion and incubated overnight at 20°C. Samples were then centrifuged at 25,000 × *g* for 45 min at 4°C, placed on ice for 5 min, and the supernatants carefully discarded. Next, 300 μL of 70% EtOH was added to the pellets and then was centrifuged at 25,000 × *g* for 10 min at 4°C. Supernatant was discarded, and the pellets were air dried for 1 h at room temperature. Finally, the pellet was resuspended in 20 L of distilled H_2O, incubated at 65°C for 1 h to resuspend the pellets, and incubated at room temperature for 1 h. Extracted DNA was then stored at 4°C until ready for second strand synthesis, library preparation, and NGS analysis.

DNase treatment

DNase digestion for NGS analysis was conducted according to Lecomte et al.[9] The 1e−11 vector genome copies, 24.2 ng lambda phage

DNA, 20 μL baseline-ZERO 10× reaction buffer, 10 μL (10 units) baseline-ZERO DNase, 8 μL of 25 mM ATP, and 4 μL (40 units) plasmid safe DNase (Biosearch Technologies) were combined with water up to a total of 200 μL. AAV +/− DNase mixture was then incubated at 37°C for 2 h. To stop the reaction for subsuquent DNA isolation, 20 μL baseline-ZERO 10× stop solution was added and incubated at 75°C for 30 min. For rAAV used in downstream transductions, reaction was stopped using the addition of 10x stop solution with no 75°C incubation step. For the −DNase condition, the reaction was the same, apart from substituting distilled H_2O for the baseline-ZERO and plasmid-safe DNases. For a negative control, only 484 ng lambda phage DNA was added to the −DNase reaction condition. For a DNase control, only 484 ng lambda phage DNA was added to the +DNase reaction condition.

For DNase treatment of rAAV to be used in transduction as opposed to NGS analysis, the reaction conditions used would need to be the same, apart from the removal of the 75°C incubation step, as this would likely denature the AAV. The sample sheet of DNase digestion of rAAV preps can be seen in Table S4.

HBB NGS library prep

HBB NGS library generation was performed as described by Lomova et al.[37]

Single-stranded virus sequencing NGS library prep

Purified DNA concentrations were quantified for each sample using Qubit fluorometric quantification. Equal amounts of input DNA were loaded into tubes and sheared to generate DNA fragments around 300 bp in size using the Bioruptor Pico sonication system (Diagenode) according to the manufacturer's instructions. Second-strand synthesis and library preparation, including adapter ligation, were performed using the SRSLY Nanoplus kit (Claret Bio) with UMI-unique dual indexes for unique molecular indexing according to the manufacturer's protocol. Importantly, to account for the heterogeneous combination of ssDNA from the rAAV genome and double-stranded contaminant DNA, this protocol, which is designed for both ssDNA and dsDNA, involved an initial denaturation step of all nucleic acid followed by second-strand synthesis and adapter ligation of all DNA molecules.

Bioinformatics analysis

Reference sequences for the rAAV genome, vector plasmid backbone, helper plasmid, and other pertinent elements were acquired in FASTA and annotated GenBank formats. These reference sequences were sourced from the public domain, where applicable.

Genome mapping utilized the ContaVect pipeline (version 0.2.1) for each FASTQ file of raw data.[10] The ContaVect pipeline incorporates a quality control module, which includes adapter trimming with the Smith-Waterman aligner (version 1.1), masking of overlapping areas in reference sequences using BLAST (version 2.10.1), and read mapping via bwa-mem (version 0.7.0).[38-40] To facilitate sample comparison regardless of sequencing depth, we computed a normalized coverage depth. This normalization involved calculating the count of reads aligned to each base (normalized per 1,000) within BAM files and dividing by the total sum of coverage for all mapped bases across the rAAV genome. We used SAMtools (version 0.1.17) to retrieve mapped and unmapped read counts and coverage.[41] SNPs were detected using MiniCaller (version 2021.07.01), which analyzed SAM files produced by the ContaVect pipeline.[42] The distribution of read density across chromosomes and mtDNA was established post-normalization to the average read coverage per sample. The cumulative percentage of alternative nucleotides (A, C, T, and G) relative to the reference was evaluated for single-nucleotide variants (SNVs). In instances where multiple variants occurred at the same nucleotide position, the variant contributions were combined. SNVs were only illustrated in graphical formats if identified in a minimum of half of all experimental samples. Unmapped reads were then targeted for BLAST search against the GenBank database to further elucidate potential contamination.[43]

DATA AND CODE AVAILABILITY

The data that support the findings of this study are available from the corresponding author upon reasonable request.

ACKNOWLEDGMENTS

The authors thank the healthy donors that provided their mobilized peripheral blood HSPCs. We thank Chi Hong Tseng of UCLA for his assistance and expertise in the statistical analysis. We thank Dr. Matteo Pellegrini and the UCLA Institute for Quantitative & Computational Biosciences for their services. The Flow Cytometry Core and the DNA Sequencing Core of the Eli & Edythe Broad Center of Regenerative Medicine and Stem Cell Research provided essential support. These studies were supported by a California Institute for Regenerative Medicine Discovery Award (DISC 12111) and the Eli & Edythe Broad Center of Regenerative Medicine and Stem Cell Research Innovation Award. C.L. was supported by the National Academy of Sciences Ford Foundation Predoctoral Fellowship.

AUTHOR CONTRIBUTIONS

Conceptualization, C.L, Z.R., and D.B.K. Methodology, C.L., Z.R., and M.M. NGS analysis, S.M.H Investigation, C.L., Z.R., and A.M. Writing – original draft, C.L. and D.B.K. Writing – review & editing, Z.R. and D.B.K. Supervision, Z.R. and D.B.K. Project administration, C.L.; Funding acquisition, D.B.K. and C. L.

DECLARATION OF INTERESTS

The authors declare no competing interests.

SUPPLEMENTAL INFORMATION

Supplemental information can be found online at https://doi.org/10.1016/j.omtm.2024.101334.

REFERENCES

1. Au, H.K.E., Isalan, M., and Mielcarek, M. (2021). Gene Therapy Advances: A Meta-Analysis of AAV Usage in Clinical Settings. Front. Med. *8*, 809118. https://doi.org/10.3389/fmed.2021.809118.

2. Rogers, G.L., Huang, C., Clark, R.D.E., Seclén, E., Chen, H.Y., and Cannon, P.M. (2021). Optimization of AAV6 transduction enhances site-specific genome editing of primary human lymphocytes. Mol. Ther. Methods Clin. Dev. *23*, 198–209. https://doi.org/10.1016/j.omtm.2021.09.003.

3. Ling, C., Bhukhai, K., Yin, Z., Tan, M., Yoder, M.C., Leboulch, P., Payen, E., and Srivastava, A. (2016). High-Efficiency Transduction of Primary Human Hematopoietic Stem/Progenitor Cells by AAV6 Vectors: Strategies for Overcoming Donor-Variation and Implications in Genome Editing. Sci. Rep. *6*, 35495. https://doi.org/10.1038/srep35495.

4. Brimble, M.A., Winston, S.M., and Davidoff, A.M. (2023). Stowaways in the cargo: Contaminating nucleic acids in rAAV preparations for gene therapy. Mol. Ther. *31*, 2826–2838. https://doi.org/10.1016/j.ymthe.2023.07.025.

5. Kimura, T., Ferran, B., Tsukahara, Y., Shang, Q., Desai, S., Fedoce, A., Pimentel, D.R., Luptak, I., Adachi, T., Ido, Y., et al. (2019). Production of adeno-associated virus vectors for in vitro and in vivo applications. Sci. Rep. *9*, 13601. https://doi.org/10.1038/s41598-019-49624-w.

6. Costa-Verdera, H., Unzu, C., Valeri, E., Adriouch, S., González Aseguinolaza, G., Mingozzi, F., and Kajaste-Rudnitski, A. (2023). Understanding and Tackling Immune Responses to Adeno-Associated Viral Vectors. Hum. Gene Ther. *34*, 836–852. https://doi.org/10.1089/hum.2023.119.

7. Chatterjee, S., Sivanandam, V., and Wong, K.K.M., Jr (2020). Adeno-Associated Virus and Hematopoietic Stem Cells: The Potential of Adeno-Associated Virus Hematopoietic Stem Cells in Genetic Medicines. Hum. Gene Ther. *31*, 542–552. https://doi.org/10.1089/hum.2020.049.

8. Sarı, T., Yüksel, M.K., Topçuoğlu, P., Tol, M., Ayyıldız, E., Özcan, M., and İlhan, O. (2010). The effect of CD34 count and clonogenic potential of hematopoietic stem cells on engraftment. Transfus. Apher. Sci. *43*, 315–320. https://doi.org/10.1016/j.transci.2010.09.020.

9. Lecomte, E., Leger, A., Penaud-Budloo, M., and Ayuso, E. (2019). Single-Stranded DNA Virus Sequencing (SSV-Seq) for Characterization of Residual DNA and AAV Vector Genomes. Methods Mol. Biol. *1950*, 85–106. https://doi.org/10.1007/978-1-4939-9139-6_5.

10. Sonoda, Y. (2021). Human CD34-negative hematopoietic stem cells: The current understanding of their biological nature. Exp. Hematol. *96*, 13–26. https://doi.org/10.1016/j.exphem.2021.02.004.

11. Ferrari, S., Jacob, A., Cesana, D., Laugel, M., Beretta, S., Varesi, A., Unali, G., Conti, A., Canarutto, D., Albano, L., et al. (2022). Choice of template delivery mitigates the genotoxic risk and adverse impact of editing in human hematopoietic stem cells. Cell Stem Cell *29*, 1428–1444.e9. https://doi.org/10.1016/j.stem.2022.09.001.

12. Romero, Z., Lomova, A., Said, S., Miggelbrink, A., Kuo, C.Y., Campo-Fernandez, B., Hoban, M.D., Masiuk, K.E., Clark, D.N., Long, J., et al. (2019). Editing the Sickle Cell Disease Mutation in Human Hematopoietic Stem Cells: Comparison of Endonucleases and Homologous Donor Templates. Mol. Ther. *27*, 1389–1406. https://doi.org/10.1016/j.ymthe.2019.05.014.

13. Schiroli, G., Conti, A., Ferrari, S., Della Volpe, L., Jacob, A., Albano, L., Beretta, S., Calabria, A., Vavassori, V., Gasparini, P., et al. (2019). Precise Gene Editing Preserves Hematopoietic Stem Cell Function following Transient p53-Mediated DNA Damage Response. Cell Stem Cell *24*, 551–565.e8. https://doi.org/10.1016/j.stem.2019.02.019.

14. Kuo, C.Y., Long, J.D., Campo-Fernandez, B., de Oliveira, S., Cooper, A.R., Romero, Z., Hoban, M.D., Joglekar, A.V., Lill, G.R., Kaufman, M.L., et al. (2018). Site-Specific Gene Editing of Human Hematopoietic Stem Cells for X-Linked Hyper-IgM Syndrome. Cell Rep. *23*, 2606–2616. https://doi.org/10.1016/j.celrep.2018.04.103.

15. Rai, P., and Malik, P. (2016). Gene therapy for hemoglobin disorders - a mini-review. J. Rare Dis. Res. Treat. *1*, 25–31.

16. Cromer, M.K., Vaidyanathan, S., Ryan, D.E., Curry, B., Lucas, A.B., Camarena, J., Kaushik, M., Hay, S.R., Martin, R.M., Steinfeld, I., et al. (2018). Global Transcriptional Response to CRISPR/Cas9-AAV6-Based Genome Editing in CD34(+) Hematopoietic Stem and Progenitor Cells. Mol. Ther. *26*, 2431–2442. https://doi.org/10.1016/j.ymthe.2018.06.002.

17. Min, S., Ji, J.H., Heo, Y., and Cho, H. (2022). Transcriptional regulation and chromatin dynamics at DNA double-strand breaks. Exp. Mol. Med. *54*, 1705–1712. https://doi.org/10.1038/s12276-022-00862-5.

18. Rogers, G.L., Martino, A.T., Aslanidi, G.V., Jayandharan, G.R., Srivastava, A., and Herzog, R.W. (2011). Innate Immune Responses to AAV Vectors. Front. Microbiol. *2*, 194. https://doi.org/10.3389/fmicb.2011.00194.

19. Silva, E., and Ideker, T. (2019). Transcriptional responses to DNA damage. DNA Repair *79*, 40–49. https://doi.org/10.1016/j.dnarep.2019.05.002.

20. Caiado, F., Pietras, E.M., and Manz, M.G. (2021). Inflammation as a regulator of hematopoietic stem cell function in disease, aging, and clonal selection. J. Exp. Med. *218*, e20201541. https://doi.org/10.1084/jem.20201541.

21. Demerdash, Y., Kain, B., Essers, M.A.G., and King, K.Y. (2021). Yin and Yang: The dual effects of interferons on hematopoiesis. Exp. Hematol. *96*, 1–12. https://doi.org/10.1016/j.exphem.2021.02.002.

22. Papanikolaou, E., Paruzynski, A., Kasampalidis, I., Deichmann, A., Stamateris, E., Schmidt, M., von Kalle, C., and Anagnou, N.P. (2015). Cell cycle status of CD34(+) hemopoietic stem cells determines lentiviral integration in actively transcribed and development-related genes. Mol. Ther. *23*, 683–696. https://doi.org/10.1038/mt.2014.246.

23. Read, J.A., Rouce, R.H., Mo, F., Mamonkin, M., and King, K.Y. (2023). Apoptosis of Hematopoietic Stem Cells Contributes to Bone Marrow Suppression Following Chimeric Antigen Receptor T Cell Therapy. Transplant. Cell. Ther. *29*, 165.e1–165.e7. https://doi.org/10.1016/j.jtct.2022.12.020.

24. Waring, P., and Müllbacher, A. (1999). Cell death induced by the Fas/Fas ligand pathway and its role in pathology. Immunol. Cell Biol. *77*, 312–317. https://doi.org/10.1046/j.1440-1711.1999.00837.x.

25. Karimian, A., Ahmadi, Y., and Yousefi, B. (2016). Multiple functions of p21 in cell cycle, apoptosis and transcriptional regulation after DNA damage. DNA Repair *42*, 63–71. https://doi.org/10.1016/j.dnarep.2016.04.008.

26. Pawlowski, J., and Kraft, A.S. (2000). Bax-induced apoptotic cell death. Proc. Natl. Acad. Sci. USA *97*, 529–531. https://doi.org/10.1073/pnas.97.2.529.

27. Idriss, H.T., and Naismith, J.H. (2000). TNF alpha and the TNF receptor superfamily: structure-function relationship(s). Microsc. Res. Tech. *50*, 184–195. https://doi.org/10.1002/1097-0029(20000801)50:3<184::Aid-jemt2>3.0.Co;2-h.

28. Negrini, M., Wang, G., Heuer, A., Björklund, T., and Davidsson, M. (2020). AAV Production Everywhere: A Simple, Fast, and Reliable Protocol for In-house AAV Vector Production Based on Chloroform Extraction. Curr. Protoc. Neurosci. *93*, e103. https://doi.org/10.1002/cpns.103.

29. Kondratov, O., Marsic, D., Crosson, S.M., Mendez-Gomez, H.R., Moskalenko, O., Mietzsch, M., Heilbronn, R., Allison, J.R., Green, K.B., Agbandje-McKenna, M., and Zolotukhin, S. (2017). Direct Head-to-Head Evaluation of Recombinant Adeno-associated Viral Vectors Manufactured in Human versus Insect Cells. Mol. Ther. *25*, 2661–2675. https://doi.org/10.1016/j.ymthe.2017.08.003.

30. Strobel, B., Miller, F.D., Rist, W., and Lamla, T. (2015). Comparative Analysis of Cesium Chloride- and Iodixanol-Based Purification of Recombinant Adeno-Associated Viral Vectors for Preclinical Applications. Hum. Gene Ther. Methods *26*, 147–157. https://doi.org/10.1089/hgtb.2015.051.

31. Giles, A., Lock, M., Chen, S.J., Turner, K., Wesolowski, G., Prongay, A., Petkov, B.N., Olagbegi, K., Yan, H., and Wilson, J.M. (2023). Significant Differences in Capsid Properties and Potency Between Adeno-Associated Virus Vectors Produced in Sf9 and HEK293 Cells. Hum. Gene Ther. *34*, 1003–1021. https://doi.org/10.1089/hum.2022.116.

32. Arjomandnejad, M., Dasgupta, I., Flotte, T.R., and Keeler, A.M. (2023). Immunogenicity of Recombinant Adeno-Associated Virus (AAV) Vectors for Gene Transfer. BioDrugs *37*, 311–329. https://doi.org/10.1007/s40259-023-00585-7.

33. Ertl, H.C.J. (2022). Immunogenicity and toxicity of AAV gene therapy. Front. Immunol. *13*, 975803. https://doi.org/10.3389/fimmu.2022.975803.

34. Ronzitti, G., Gross, D.A., and Mingozzi, F. (2020). Human Immune Responses to Adeno-Associated Virus (AAV) Vectors. Front. Immunol. *11*, 670. https://doi.org/10.3389/fimmu.2020.00670.

35. Zhang, J., Guo, P., Yu, X., Frabutt, D.A., Lam, A.K., Mulcrone, P.L., Chrzanowski, M., Firrman, J., Pouchnik, D., Sang, N., et al. (2022). Subgenomic particles in rAAV vectors result from DNA lesion/break and non-homologous end joining of vector genomes. Mol. Ther. Nucleic Acids *29*, 852–861. https://doi.org/10.1016/j.omtn.2022.08.027.

36. Radtke, S., Humbert, O., and Kiem, H.P. (2020). Mouse models in hematopoietic stem cell gene therapy and genome editing. Biochem. Pharmacol. *174*, 113692. https://doi.org/10.1016/j.bcp.2019.113692.

37. Lomova, A., Clark, D.N., Campo-Fernandez, B., Flores-Bjurström, C., Kaufman, M.L., Fitz-Gibbon, S., Wang, X., Miyahira, E.Y., Brown, D., DeWitt, M.A., et al. (2019). Improving Gene Editing Outcomes in Human Hematopoietic Stem and Progenitor Cells by Temporal Control of DNA Repair. Stem Cells *37*, 284–294.

OMTM, Volume *32*

Supplemental information

DNA contamination within recombinant adeno-associated virus preparations correlates with decreased $CD34^+$ cell clonogenic potential

Christopher R. Luthers, Sung-Min Ha, Annika Mittelhauser, Marco Morselli, Joseph D. Long, Caroline Y. Kuo, Zulema Romero, and Donald B. Kohn

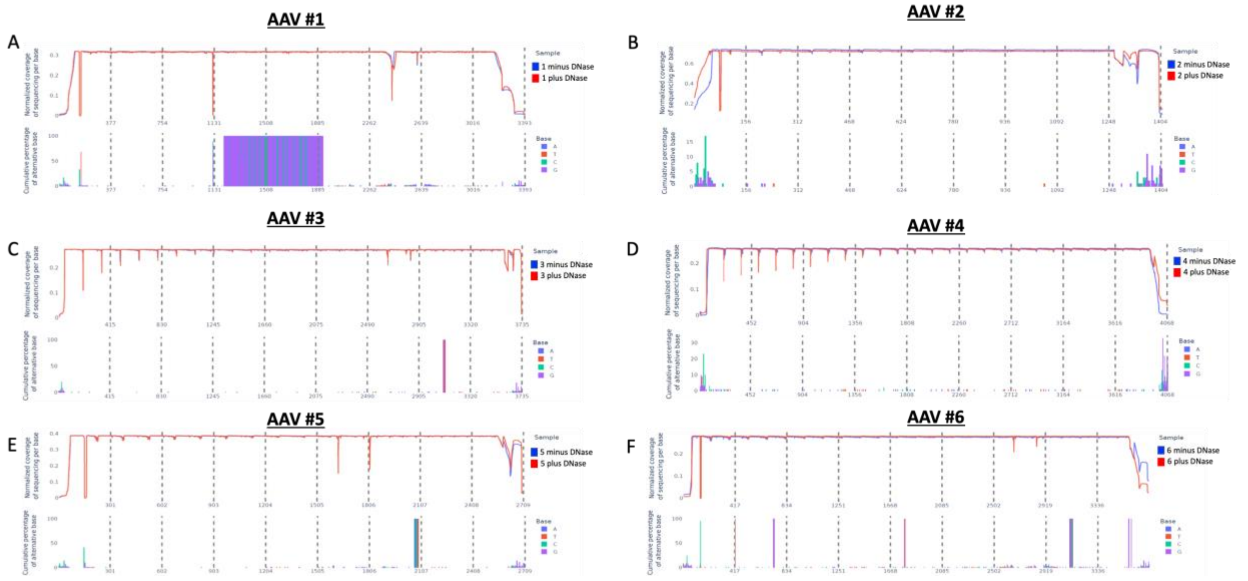

Figure S1: Read Coverage and Single Nucleotide Variants Across the AAV Genome. (Top) Coverage of sequencing reads across the rAAV genome was normalized by dividing the number of reads aligned to each base and diving that by the total coverage of all bases which mapped to the AAV genome. (Bottom) Total percentage of alternative nucleotide variants (A=blue, T=red, C=green, G=purple) uncovered from each base call compared to reference rAAV genomic sequences. (A)=AAV #1, (B)=AAV #2, (C)=AAV #3, (D)=AAV #4, (E)=AAV #5, (F)=AAV #6

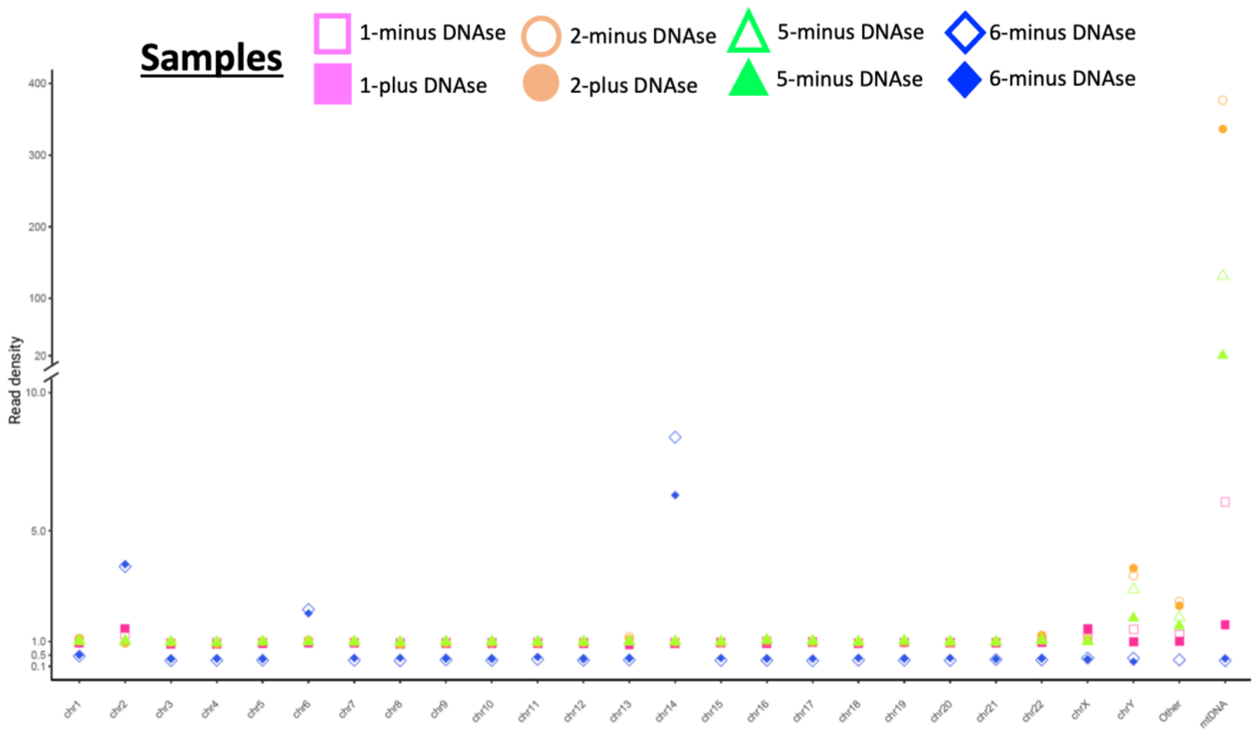

Figure S2: Distribution of Genomic DNA Contaminants Across Human Genome Chromosomes. For the four preps which were packaged using human cells, read depth of contaminants from genomic DNA was mapped to each human chromosome. Read density of 1=random distribution, read density 2 = 2 fold enrichment**.**

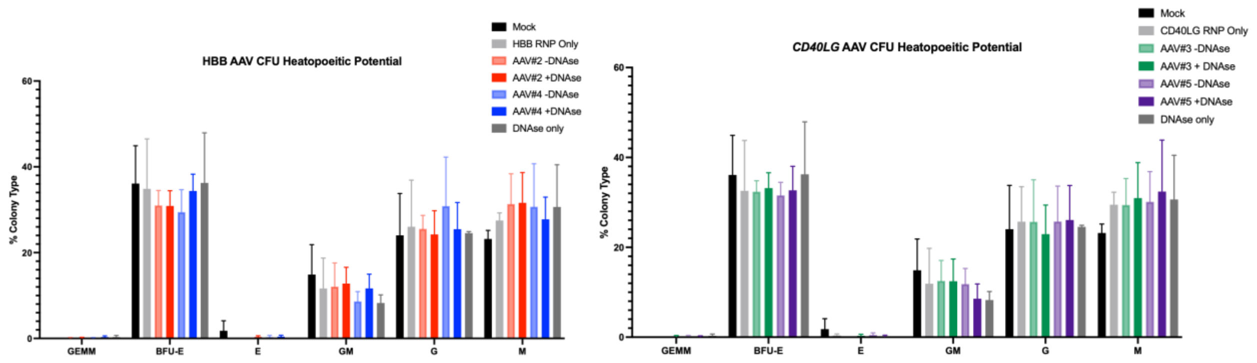

Figure S3: Progenitor analysis of CFU assay. At 24 h post-EP, PBSCs were harvested, counted by trypan blue exclusion, and plated in methylcellulose to perform the colony-forming unit (CFU) assay. At 12–14 days after plating the cells in methylcellulose, colonies were enumerated. For each plate, the percentage of cells growing was obtained as the total number of colonies counted normalized to the number of cells plated per dish for rAAV6 preps targeting the HBB (A) and CD40LG (B) loci, respectively. n = 3 independent experiments from three different PBSC donors. Error bars, mean ± SD.

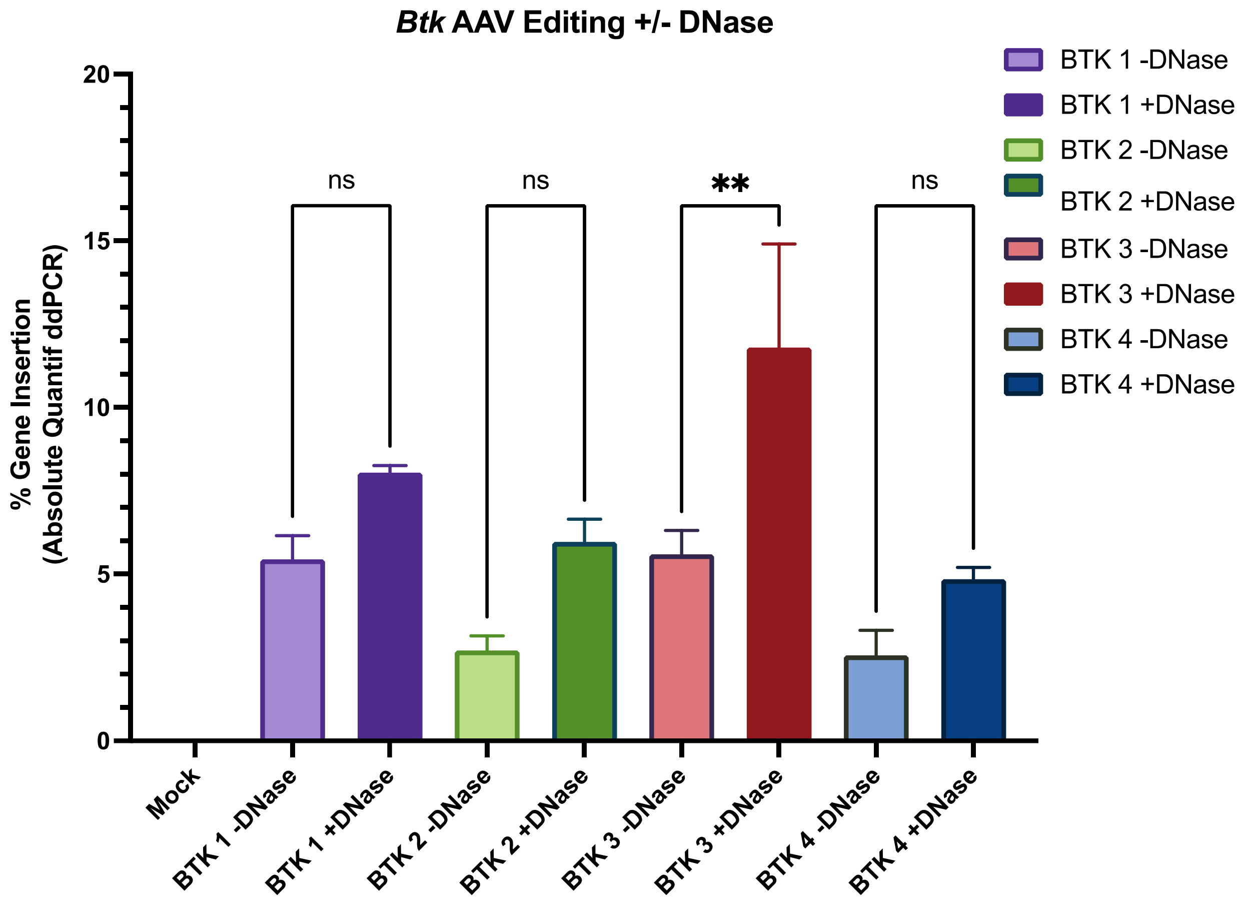

Figure S4: DNAse pretreatment increases editing in alternative cell types and genomic loci. Bone marrow derived lineage depleted Lin- cells HSPC were harvested from Btk/Tec-/- mice cells. Following 48-hour prestimulation, lin- cells were then electroporated with Cas9-RNP targeting the endogenous *Btk* locus and transduced with 4 rAAV6 vectors +/- DNAse treatment (Table 1). 5 days post transduction, gDNA of Lin- cells was harvested followed by droplet digital PCR analysis of *Btk* site-specific editing analysis. Values indicate the percentage of DNA sequences with successful insertion of corrective *Btk* cDNA sequences. N=3 independent murine Lin- transductions.

Table S1: Read Quality and Depth of NGS Analysis. PHRED quality scores and number of reads for each NGS run analyzed. “Treatment” refers to absence or presence of DNAse treatment of the rAAV6 preps.

Sample Name	Treatment	Mean read quality	Number of reads
1-minus	-	27.61	8,499,170
		27.72	8,499,170
1-plus	+	28.44	9,938,328
		27.67	9,938,328
2-minus	-	28.66	30,718,308
		27.50	30,718,308
2-plus	+	28.50	24,433,069
		27.56	24,433,069
3-minus	-	28.19	21,862,350
		26.99	21,862,350
3-plus	+	27.47	25,443,374
		26.74	25,443,374
4-minus	-	28.04	5,787,053
		26.85	5,787,053
4-plus	+	28.57	23,258,440
		27.12	23,258,440
5-minus	-	27.66	10,846,160
		26.89	10,846,160
5-plus	+	27.85	6,195,556
		27.09	6,195,556
6-minus	-	26.49	3,920,919
		27.77	3,920,919
6-plus	+	26.33	2,142,433
		26.68	2,142,433

Table S2: Comparing the Read Distribution of ITR for NGS Analysis in Merged AAV-Vector Backbone Reference Sequences. To further analyze the read quality and distribution of NGS reads across the ITR, Contavect analysis was conducted using two methods: one with a merged reference sequence containing the entire vector plasmid (AAV + ITR sequences) or using unique reference sequences for the rAAV genome and AAV plasmid backbone sequence. Values shown above are raw number of reads in the ITR's, the AAV sequence, and the percentage of total reads which come from the ITR.

	Separated AAV and Backbone references			Merged AAV and Backbone references		
	reads in ITR	reads in AAV	% reads in ITR	reads in ITR	reads in AAV	% reads in ITR
1-minus	340,316	5,495,240	4.28%	344,342	5,495,222	4.33%
	351,381	5,347,083	4.42%	356,109	5,347,032	4.47%
1-plus	523,613	8,414,450	5.5%	531,204	8,415,850	5.58%
	544,833	8,331,851	5.73%	554,020	8,334,639	5.82%
2-minus	7,403	327,111	0.02%	13,314	343,967	0.04%
	8,034	321,643	0.03%	13,525	337,355	0.05%
2-plus	5,529	282,079	0.02%	12,344	282,189	0.05%
	5,506	279,138	0.02%	12,309	279,200	0.05%
3-minus	611,739	16,912,460	2.96%	619,981	16,909,716	3%
	330,099	16,429,882	1.6%	346,920	16,429,493	1.68%
3-plus	883,705	22,695,634	3.72%	892,696	22,682,335	3.76%
	395,254	20,938,698	1.67%	412,161	20,938,388	1.74%
4-minus	101,523	5,311,944	1.84%	38,930	5,291,672	0.71%
	75,475	5,248,768	1.37%	40,588	5,245,309	0.74%
4-plus	287,258	21,713,704	1.27%	144,689	21,652,884	0.64%
	302,498	21,185,477	1.34%	155,085	21,165,785	0.69%
5-minus	130,254	4,687,617	1.28%	134,373	4,689,318	1.32%
	197,388	4,451,614	1.94%	204,585	4,452,895	2.01%
5-plus	144,484	4,920,014	2.5%	148,053	4,921,760	2.57%
	218,092	4,752,140	3.78%	224,893	4,753,502	3.9%
6-minus	43,815	2,659,202	1.22%	43,960	2,659,247	1.22%
	38,888	2,315,550	1.08%	39,087	2,315,603	1.09%
6-plus	26,946	1,663,936	1.41%	27,021	1,663,961	1.41%
	25,801	1,556,942	1.35%	25,926	1,556,994	1.35%

Table S3: Absolute Read Numbers of DNA Products Within AAV Preps. The amount of reads for each DNA contaminant as they map to known contaminant sequences. Numbers represent total individual reads.

Reference name	1-minus	1-plus	2-minus	2-plus	5-minus	5-plus	6-minus	6-plus
Phi X174	2	4	12	4	1	1	3	2
rAAV genome	10,858,317	16,767,045	649,015	561,952	9,146,508	9,680,613	4,979,995	3,223,010
Plasmid backbone	440,435	748,995	128,276	100,832	132,442	95,575	33,515	19,737
Helper plasmid	479,620	814,580	782,540	741,113	252,222	134,868	235,163	139,504
Human genome	312,984	74,242	51,800,765	40,761,803	5,368,041	996,459	33,695	18,260
Vigene Rep Cap	25,633	48,246	197,455	166,776	48,399	18,266	11,890	6,760
Unmappe d	3,798,663	576,216	6,389,869	5,422,208	5,368,749	611,770	1,895,955	422,821
Total	15,915,654	1,902,9328	59,947,932	47,754,688	20,316,362	11,537,552	7,190,216	3,830,094

Reference name	3-minus	3-plus	4-minus	4-plus
Phi X174	18	17	1	6
rAAV genome	33,356,896	43,648,328	10,560,924	42,901,282
Plasmid backbone	0	0	0	0
Full plasmid	317,144	324,340	14,195	74,387
Baculovirus	83,027	73,175	32,192	188,441
Virovek Rep Cap	1,904	1,069	570	2,979
Sf-9	27,108	24,599	24,576	89,726
Unmapped	7,520,875	3,377,918	406,552	1,971,951
Total	41,306,972	47,449,446	11,039,010	45,228,772

Table S4. Example Table for DNase Treatment of rAAV6 Vectors. Sample calculation sheet for pretreatment of rAAV

			DNAse Calculation						
**Condition**	**uL AAV**	**uL ATP**	**uL Baseline DNAse**	**uL Plasmid safe DNAse**	**uL H20**	**Total Volume**	**uL 10x buffer**	**Total uL before STOP**	**uL 10x STOP**
**2 +DNase**	10.83	20	10	4	0	44.83	4.48	49.32	4.93
**2 -DNase**	10.83	20	0	0	14.00	44.83	4.48	49.31	4.93
**4 +DNase**	6.16	20	10	4	4.67	44.83	4.48	49.31	4.93
**4 -DNase**	6.16	20	0	0	18.67	44.83	4.48	49.31	4.93
**3 +DNase**	3.23	20	10	4	7.60	44.83	4.48	49.31	4.93
**3 -DNase**	3.23	20	0	0	21.60	44.83	4.48	49.31	4.93
**5 +DNAse**	4.92	20	10	4	5.91	44.83	4.48	49.31	4.93
**5 -DNAse**	4.92	20	0	0	19.91	44.83	4.48	49.31	4.93
**DNAse only**	0.00	20	10	4	10.83	44.83	4.48	49.31	4.93
